# Enhancing H_2_ evolution performance of an immobilised cobalt catalyst by rational ligand design†
†Electronic supplementary information (ESI) available: Additional figures and tables, synthetic procedures, experimental details for NMR and UV-vis spectroscopy, electrochemistry and photocatalytic experiments. See DOI: 10.1039/c4sc03946g



**DOI:** 10.1039/c4sc03946g

**Published:** 2015-02-02

**Authors:** Janina Willkomm, Nicoleta M. Muresan, Erwin Reisner

**Affiliations:** a Christian Doppler Laboratory for Sustainable SynGas Chemistry , Department of Chemistry , University of Cambridge , Lensfield Road , Cambridge CB2 1EW , UK . Email: reisner@ch.cam.ac.uk ; http://www-reisner.ch.cam.ac.uk/

## Abstract

The catalyst [Co^III^Br((DO)(DOH)(4-BnPO_3_H_2_)(2-CH_2_py)pn)]Br, **CoP^3^**, has been synthesised to improve the stability and activity of cobalt catalysts immobilised on metal oxide surfaces. The **CoP^3^** catalyst contains an equatorial diimine–dioxime ligand, (DOH)_2_pn = *N*^2^,*N*^2^′-propanediyl-bis(2,3-butanedione-2-imine-3-oxime), with a benzylphosphonic acid (4-BnPO_3_H_2_) group and a methylpyridine (2-CH_2_py) ligand covalently linked to the bridgehead of the pseudo-macrocyclic diimine–dioxime ligand. The phosphonic acid functionality provides a robust anchoring group for immobilisation on metal oxides, whereas the pyridine is coordinated to the Co ion to enhance the catalytic activity of the catalyst. Electrochemical investigations in solution confirm that **CoP^3^** shows electrocatalytic activity for the reduction of aqueous protons between pH 3 and 7. The metal oxide anchor provides the catalyst with a high affinity for mesostructured Sn-doped In_2_O_3_ electrodes (*meso*ITO; loading of approximately 22 nmol cm^–2^) and the electrostability of the attached **CoP^3^** was confirmed by cyclic voltammetry. Finally, immobilisation of the catalyst on ruthenium-dye sensitised TiO_2_ nanoparticles in aqueous solutions in the presence of a hole scavenger establishes the activity of the catalyst in this photocatalytic scheme. The advantages of the elaborate catalyst design in **CoP^3^** in terms of stability and catalytic activity are shown by direct comparison with previously reported phosphonated Co catalysts. We therefore demonstrate that rational ligand design is a viable route for improving the performance of immobilised molecular catalysts.

## Abstract 2

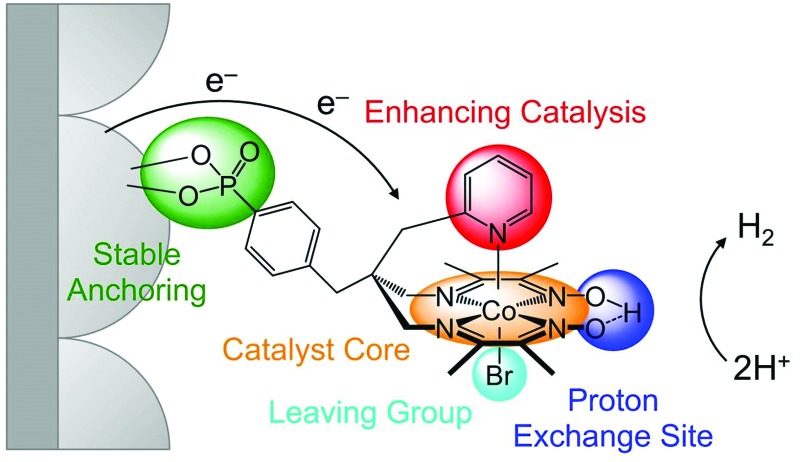
Rational ligand design was employed to improve the proton reduction activity of an immobilised cobalt diimine–dioxime catalyst.

## Introduction

Solar fuels generation through artificial photosynthesis requires a well-balanced combination of light harvesting and charge separation with proton reduction and water oxidation catalysis, preferentially in a photoelectrochemical (PEC) cell.^1^ As for H_2_ evolution, molecular synthetic catalysts based on 3d transition metals like Fe,^2^ Co^3^ or Ni^4^ are currently under intensive investigation as an alternative to the current benchmark H_2_ evolving catalysts: scarce and expensive Pt^5^ and fragile enzymes known as hydrogenases.^6^ However, the use of catalysts in a PEC cell requires their stable integration into electrodes, which is particularly challenging for molecular catalysts.^7^

An advantage of synthetic molecular catalysts compared to solid-state materials or enzymes is the relative ease to control and characterise their composition and to study their mechanisms and kinetics in great detail. This strength provides a rational route to elaborated and improved catalyst design through mechanistic understanding and often by adopting hydrogenase-related principles.^8^ For example, bio-inspired nickel bis(diphosphine) catalysts were reported to generate H_2_ photo-^9^ and electrocatalytically^9,10^ in aqueous solution. These Ni complexes remain electroactive when heterogenised on carbon-based electrodes,^11^ and immobilisation on metal oxide nanoparticles^9^ and on carbon nitride^12^ has allowed for their exploitation for photocatalytic H_2_ production in heterogeneous schemes. Synthetic mimics of the [FeFe]-hydrogenase active site evolve H_2_ from water when combined with CdTe quantum dots as a photosensitiser^13^ and when incorporated into a protective environment, *e.g.* a metal organic framework^14^ or a micellar system.^15^

**Fig. 1:**
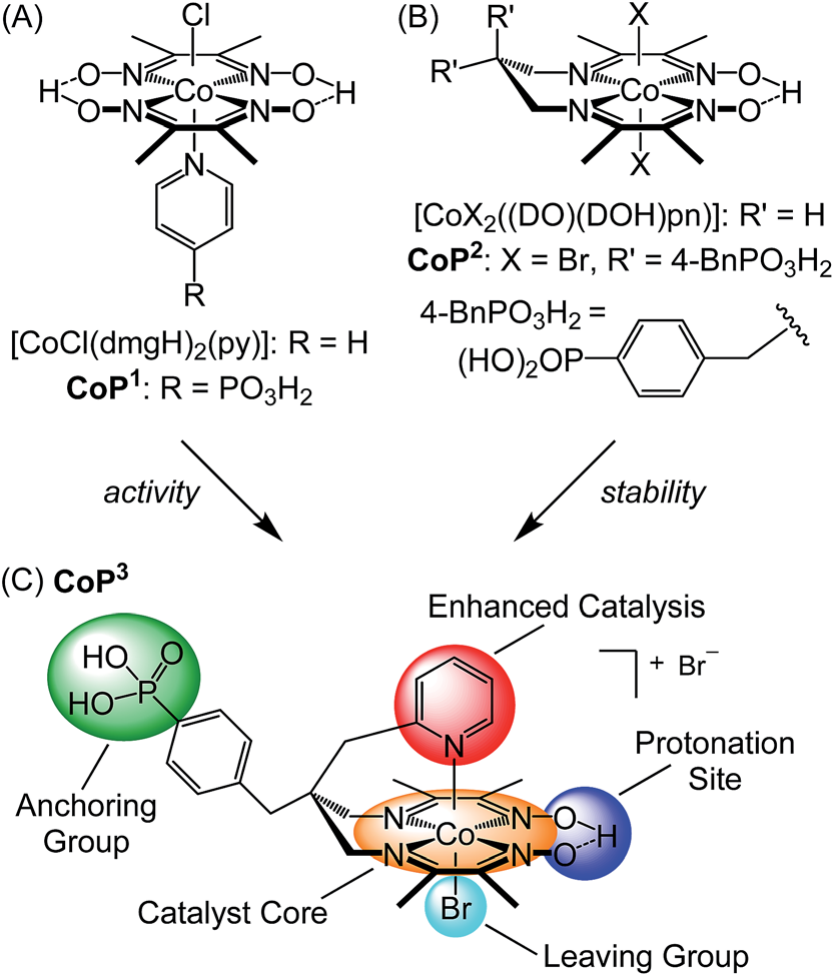
Chemical structures of (A) cobaloximes with an axial pyridine ligand, (B) cobalt diimine–dioxime catalysts, and (C) catalyst **CoP^3^** reported in this study. **CoP^3^** was designed to incorporate the activity enhancing pyridine of **CoP^1^** (A),^20*b*^ and the stable catalyst core and anchoring functionality of **CoP^2^** (B).^24^

Cobalt catalysts with a bis(dimethylglyoximato) equatorial ligand (dmgH^–^)_2_ and an activity enhancing axial pyridine ligand,^3h,16^ [CoCl(dmgH)_2_(py)] (Fig. 1A), have long been identified as one of the most active molecular catalysts for the reduction of aqueous protons and a wealth of experimental and theoretical information is available.^17^ These catalysts belong to the class of cobaloximes and they are also among the very few synthetic catalysts reported as O_2_-tolerant during catalysis, which is an important consideration for their use in full water splitting systems.^16a,18^ Cobaloximes have been integrated into photocatalytic systems by wiring the catalyst to a light absorber. For example, supramolecular homogeneous systems with a dye covalently linked to the Co catalyst,^19^ colloidal systems containing dye-sensitised titania (with **CoP^1^**, R = PO_3_H_2_; Fig. 1A)^20^ or carbon nitride^21^ and their immobilisation on photocathodes^7b,22^ have been reported. However, these assemblies suffer from the drawback of anchoring the cobaloxime to the light absorber *via* the monodentate axial pyridine ligand. The Co–pyridine bond becomes labile during catalysis, which may result in the loss of the Co(dmgH)_2_ core from the light absorber unit during irradiation.^19a,23^ Consequently, the stability and performance of these photocatalytic systems are limited.

A more robust class of cobalt catalysts, [CoX_2_((DO)(DOH)pn)] with X = bromide or chloride and the tetradentate ligand (DOH)_2_pn = *N*^2^,*N*^2^′-propanediyl-bis(2,3-butanedione-2-imine-3-oxime) (R′ = H; Fig. 1B),^3d,3i,25^ was recently integrated into electrodes. This Co catalyst was immobilised on a carbon-based electrode *via* click chemistry (X = Cl, R′ = H, N_3_)^26^ and on a conducting metal oxide electrode *via* a phosphonic acid linker (**CoP^2^**, X = Br, R′ = 4-BnPO_3_H_2_; Fig. 1B).^24,27^ Anchoring of the Co catalyst through the propanediyl bridgehead of the pseudo-macrocyclic equatorial ligand provides a substantially more stable anchoring to an electrode than immobilisation *via* the axial pyridine in cobaloximes.

In this work, we present a cobalt catalyst for H_2_ evolution, which does not only display good stability when anchored onto metal oxide surfaces, but also enhanced catalytic activity compared to the previously reported immobilised Co catalyst **CoP^2^**. The novel cobalt catalyst, **CoP^3^**, contains a pendant pyridine and a dangling phosphonic acid group linked to the bridgehead of the equatorial diimine–dioxime ligand (Fig. 1C). The axial pyridine ligand coordinates to the metal centre and enhances the activity of the cobalt catalyst. Covalent linkage to the equatorial ligand framework ensures that the pyridine does not diffuse away from the catalyst core during turnover. The phosphonic acid group allows for attachment to metal oxide surfaces and is also tightly bound to the ligand framework. The electrochemistry of **CoP^3^** in solution and when immobilised on mesoporous indium–tin oxide electrodes (ITO|*meso*ITO), as well as the photocatalytic activity of **CoP^3^** in Ru-dye sensitised systems is reported and the results are directly compared with previously reported cobalt catalysts **CoP^1^** and **CoP^2^** (Fig. 1).

## Results and discussion

### Synthesis and characterisation of CoP^3^

**Scheme 1:**
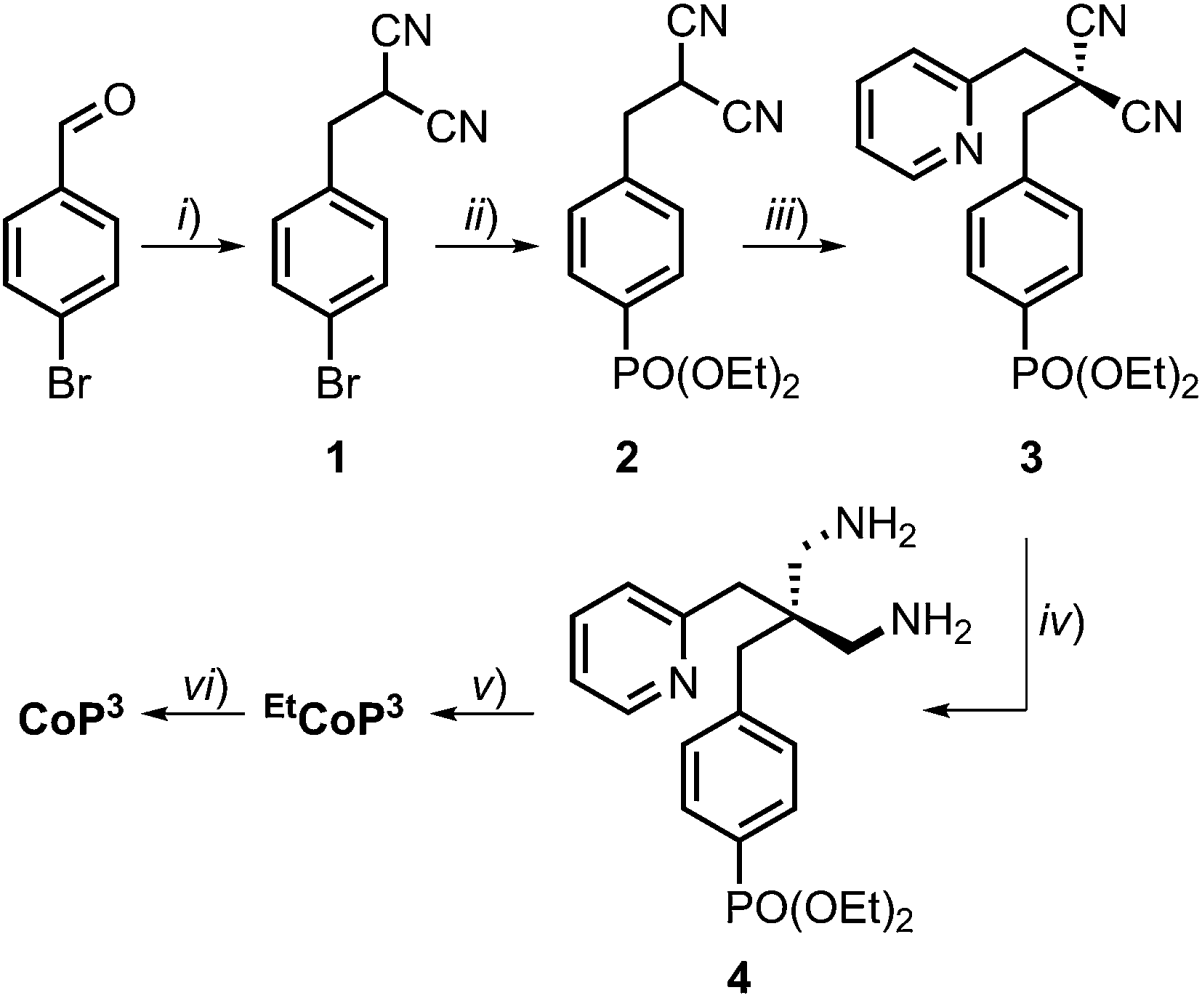
(i) Malononitrile, NaBH_4_, ethanol/water (95/5), 3 h, r.t., 80%; (ii) HPO(OEt)_2_, Et_3_N, Pd(PPh_3_)_4_, PPh_3_, tetrahydrofuran, 48 h, reflux, 73%; (iii) 2-(bromomethyl)pyridine·HBr, K_2_CO_3_, acetone, 3d, r.t., 58%; (iv) borane, tetrahydrofuran, 24 h, r.t., 99%; (v) 2,3-butanedione monoxime, CoBr_2_·6H_2_O, air, methanol, 5d, r.t., 45%; (vi) bromotrimethylsilane, dichloromethane, 48 h, r.t., 65% yield. The chemical structure of **CoP^3^** is shown in Fig. 1C.

Complex **CoP^3^** was synthesised in six steps from commercially available starting materials with an overall yield of approximately 10% (Scheme 1 and ESI† for experimental details).

Compound **1** was prepared *via* condensation of 4-bromobenzaldehyde with malononitrile and reduction by NaBH_4_.^28^ The phosphonate ester derivative **2** was synthesised from **1** in a Pd-catalysed cross-coupling reaction with diethyl phosphite. Introduction of the pendant pyridine was achieved by alkylation of **2** with 2-(bromomethyl)pyridine. The resulting malononitrile derivative **3** was reduced to the diamine **4** by treatment with borane. Complex **^Et^CoP^3^** was obtained from a one-pot, three-step condensation–complexation–oxidation reaction:^24,25c^ the diimine–dioxime ligand was prepared *via* condensation of **4** and 2,3-butanedione monoxime, followed by addition of CoBr_2_·6H_2_O and oxidation of the Co^II^ ion in air to form **^Et^CoP^3^**. Hydrolysis of the phosphonate ester using bromotrimethylsilane yielded the target complex **CoP^3^**. ^1^H, ^13^C and ^31^P NMR spectra of the compounds are shown in Fig. S1 to S11.†

The final complex **CoP^3^** was characterised by ^1^H, ^13^C, ^31^P and NOE NMR spectroscopy, UV-vis and ATR-IR spectroscopy, mass spectrometry and elemental analysis. The ^31^P NMR spectra of the phosphonate ester compounds **2–4** and **^Et^CoP^3^** feature a signal at approximately 19 ppm, which is shifted to 13 ppm in **CoP^3^** as expected upon hydrolysis of the phosphonate ester. Both cobalt complexes, **^Et^CoP^3^** and **CoP^3^** display a characteristic ^1^H NMR signal at approximately 19 ppm, which is assigned to the bridge proton of the equatorial (DO)(DOH)pn ligand.^24,29^ ^1^H NMR signals of the methylene protons on the propanediyl bridgehead of diamine **4** exhibit a downfield shift from 2.5 ppm to 3.7 and 4.1 ppm upon formation of the cobalt diimine–dioxime complex **^Et^CoP^3^**. Moreover, these diastereotopic methylene protons (^2^*J*(H,H) = 15 Hz) show a significantly different chemical shift (for **CoP^3^**: Δ*δ* = 0.6 ppm in DMSO-*d*_6_). This difference is presumably due to two different axial ligands in the octahedral coordination sphere and is an indication of coordination of the pendant pyridine ligand to the metal centre in **^Et^CoP^3^** and **CoP^3^**. Evidence for coordination is also given by a 0.7 ppm upfield shift of the signal of the pyridine proton in 6-position upon formation of the cobalt complexes (H6, Table S1†).^29^ In addition, a NOE response was observed for this proton after saturation of the oxime proton signal at 19.2 ppm (Fig. S12†) revealing that both protons have to be in close proximity to each other.^29^ When trifluoroacetic acid (TFA) was added to a solution of **CoP^3^** in DMSO-*d*_6_, no shift of the pyridine proton signals was observed (Fig. S13†). If protonated, new signals would be expected in the range of 8 to 9 ppm.^30^ Thus, the pyridine remains ligated to the cobalt centre and is not protonated even in the presence of a strong acid.

The ^1^H NMR spectrum of **CoP^3^** in D_2_O shows a similar upfield shift for the pyridine proton in 6-position as in DMSO-*d*_6_ (7.8 ppm in **CoP^3^***vs.* 8.5 ppm in diamine **4**) and the spectrum remained unchanged for at least three weeks (Fig. S14†). Electronic absorption spectra of **CoP^3^** in water show a strong π–π* absorption at *λ* = 259 and 219 nm (*ε* = 1.864 × 10^4^ L mol^–1^ cm^–1^ and 2.774 × 10^4^ L mol^–1^ cm^–1^; Fig. S15†). Similar absorption features are obtained in pH 7 phosphate buffer and pH 4.5 acetate buffer and no changes in the UV-vis spectrum were apparent when the solution was acidified with TFA (Fig. S15†), demonstrating the good stability of the catalyst in aqueous solutions.

### Electrochemical studies in solution

The electrochemical response of **CoP^3^** was investigated in organic as well as aqueous electrolyte solutions using a three-electrode set-up with a glassy carbon working electrode (0.07 cm^2^). A cyclic voltammogram (CV) of **CoP^3^** recorded in DMF/TBABF_4_ electrolyte solution (TBABF_4_ = tetrabutylammonium tetrafluoroborate, 0.1 M) exhibits two reversible one-electron reduction waves at *E*_1/2_ = –0.67 V and –1.07 V *vs.* Fc^+^/Fc, which are assigned to the Co^III^/Co^II^ and Co^II^/Co^I^ redox couples, respectively (Fig. S16A†).^3i,24^ Upon addition of 1 to 10 equivalents of TFA, a catalytic proton reduction wave appeared close to the potential of the initial Co^II^/Co^I^ redox couple at a half-wave potential, *E*_cat/2_, of –1.06 V *vs.* Fc^+^/Fc, (Fig. S16B†). Thus, an overpotential (*η*) of approximately 110 mV is required to reduce TFA protons (*E*^0^(H^+^/H_2_) = –0.95 V *vs.* Fc^+^/Fc for 10 mM TFA in DMF)^31^ with **CoP^3^**, which is comparable to previously reported [Co(DO)(DOH)pn]-type complexes.^3i,24^

**Fig. 2:**
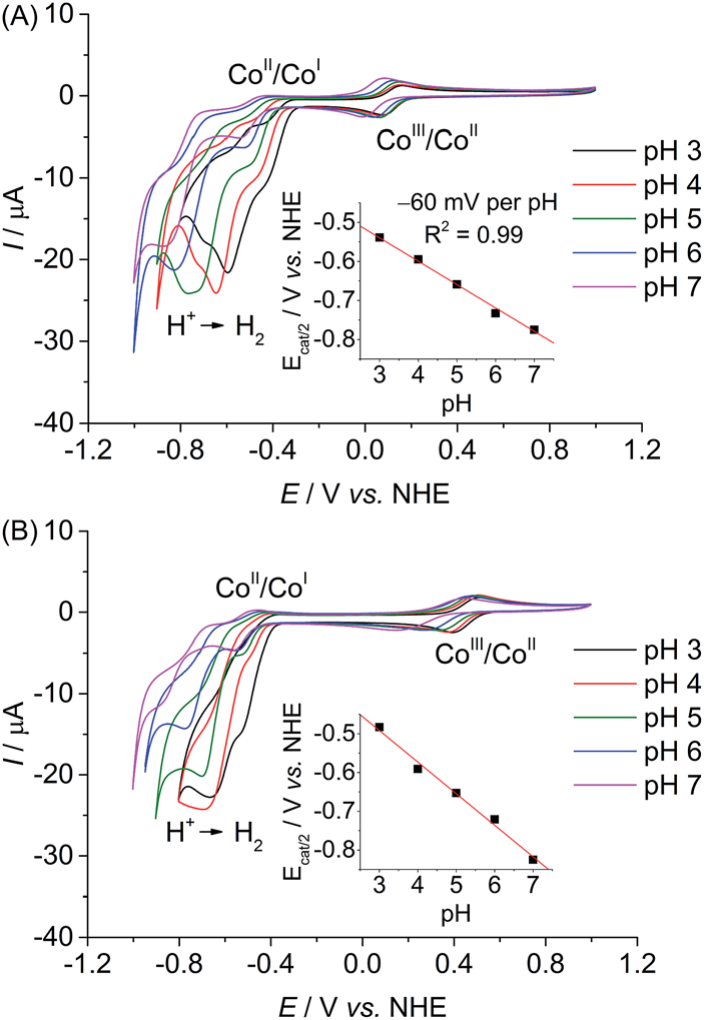
CVs with dissolved (A) **CoP^3^** and (B) **CoP^2^** (0.8 mM) recorded in an aqueous Britton–Robinson buffer at different pH values on a glassy carbon working electrode at 20 mV s^–1^. The insets show the correlation between the half-wave potential of the catalytic reduction wave, *E*_cat/2_, and the pH value. The red traces represent the linear fit of the data points.

CVs recorded in aqueous Britton–Robinson buffer (pH 3 to 7) feature a reversible Co^III^/Co^II^ redox couple and quasi-reversible Co^II^/Co^I^ reduction (Fig. 2A). When scanning towards more cathodic potential, a third reduction wave is observed which is attributed to catalytic proton reduction by the complex.^3*d*^ Comparable electrochemical responses were obtained when a pH 7 triethanolamine (TEOA)/Na_2_SO_4_ electrolyte solution and pH 4.5 acetate or ascorbic acid (AA) solution were used (Fig. S17†), except that no Co^III^/Co^II^ reduction wave can be observed in cathodic scans in AA solution, presumably due to the chemical reduction of **Co^III^P^3^** to **Co^II^P^3^** (Fig. S18†). The onset of a weak wave, tentatively assigned to Co^II^/Co^III^ oxidation, is observed at approximately 0.05 V *vs.* NHE before AA oxidation starts at 0.2 V *vs.* NHE.

The pH-dependent investigation also revealed that the half-wave potential of the catalytic reduction wave of **CoP^3^**, *E*_cat/2_, shifts by approximately –60 mV per pH unit increase (Fig. 2A); in agreement with a one proton–one electron coupled process according to the Nernst equation. This was previously attributed to protonation of the oxime functionality in [Co(DO)(DOH)pn]-type complexes.^3i,25a^

Comparison of the electrochemical response of **CoP^3^** to the previously reported complex **CoP^2^** allows us to elucidate any beneficial effect of the additional axial pyridine ligand on the proton reduction activity. CVs of **CoP^2^** recorded in the pH range from 3 to 7 are shown in Fig. 2B. A shift in redox potential is observed for the Co^III^/Co^II^ redox couple in **CoP^3^** compared to **CoP^2^** (Δ*E*_1/2_ = –0.24 V at pH 7), which is consistent with a coordinated pyridine in **CoP^3^**. For both cobalt diimine–dioxime catalysts, the catalytic reduction wave decreases with increasing pH indicating a higher proton reduction activity under more acidic conditions, which has been previously observed for (DO)(DOH)pn-type cobalt catalysts.^26^ Peak currents of the catalytic reduction wave, *I*_cat_, and *I*_cat_/*I*_p_ ratios taking into account the non-catalytic Co^III^/Co^II^ reduction peak currents, *I*_p_, are similar for both complexes at pH 3 and 4 (Table S2†). But, **CoP^3^** features higher *I*_cat_ and *I*_cat_/*I*_p_ ratios at pH values above 4 revealing a higher activity of **CoP^3^** under more pH neutral conditions (Table S2†). Moreover, the half wave potential *E*_cat/2_ of **CoP^3^** is observed at less negative potentials than for **CoP^2^** under pH neutral conditions (–0.83 V for **CoP^2^***vs.* –0.78 V *vs.* NHE for **CoP^3^**).

The half-wave potential, *E*_1/2_, of the Co^II^/Co^I^ reduction wave in **CoP^3^** shifts with about –33 mV per pH at pH values below 6 and becomes almost pH independent above pH 6 (Fig. S19A†). Such a change in slope was not observed for *E*_1/2_(Co^II^/Co^I^) in **CoP^2^** (Fig. S19B†), suggesting an alteration in the coordination sphere specific to **CoP^3^**, *e.g.* a ligated and non-ligated, probably protonated pendant pyridine ligand. The pH-dependencies of *E*_1/2_ of the Co^III^/Co^II^ reduction wave change in a similar manner for **CoP^2^** and **CoP^3^** (Fig. S20†) and are ascribed to protonation/deprotonation occurring at moieties present in both complexes, *e.g.* at phosphonic acid groups^9^ or aquo ligands. Due to a different number of those functionalities the slopes differ for both complexes.

Based on these findings, we suggest that the enhanced catalytic activity of **CoP^3^** under near neutral conditions is due to coordination of the pyridine to the cobalt centre during the catalytic cycle. The electron donating ability of the pyridine ligand would allow for the formation of a more basic Co-hydride species in the rate limiting step of the catalytic cycle, thereby improving proton reduction catalysis.^16a,32^ A similar increase of catalytic current and decrease in overpotential has previously been observed when an axial pyridine ligand was introduced to the coordination sphere of cobaloxime complexes at neutral pH.^16*a*^ Addition of one and four equivalents of pyridine to a **CoP^2^**-containing electrolyte solution at pH 7 did not result in any increase of the catalytic reduction wave, which demonstrates that the covalent integration of the pyridine as achieved in **CoP^3^** is also critical to enhance the activity of the cobalt diimine–dioxime catalyst (Fig. S21†).^25*c*^

The comparable pH-dependent shifts of *E*_1/2_(Co^II^/Co^I^) for **CoP^2^** and **CoP^3^** below pH 6 suggest a temporary non-coordinated pyridine in **CoP^3^** upon reduction. Although the axial pyridine in **CoP^3^** is coordinated to the cobalt centre in the initial Co^III^ state even in the presence of a strong acid (see above), reduction to Co^II^ or a formal Co^I^ species results in a labile Co–pyridine bond and subsequent release of the pyridine from the Co ion. However, the covalently linked pyridine ligand remains in close proximity to the cobalt centre and could improve catalysis in two distinct ways. It could be partially protonated under acidic conditions (p*K*_a_ of 2-picoline: 5.96)^33^ and consequently act as a proton relay in the catalytic cycle or it could readily re-coordinate and enhance activity as described above.^16*a*^ The fully reversible Co^III^/Co^II^ redox couple indicates that the pyridine re-coordinates to the Co centre upon oxidation of the complex.

Finally, both Co diimine–dioxime catalysts were compared to the phosphonated cobaloxime catalyst **CoP^1^**. Among the series of phosphonated cobalt catalyst, **CoP^1^** is the most active proton reduction catalyst at neutral pH, featuring a large proton reduction wave at more positive potential than **CoP^2^** and **CoP^3^** (Fig. S17A†). Under more acidic conditions, no Co^II^ to Co^III^ oxidation wave was observed for **CoP^1^** in the anodic reverse scans (Fig. S17B and S18A†) indicating catalyst decomposition due to hydrolysis of the equatorial (dmgH^–^)_2_ ligand.^34^

### Electrochemical studies with heterogenised catalysts

The phosphonic acid anchoring groups in **CoP^*n*^** (*n* = 1 to 3) allow for the grafting of the complexes onto metal oxide surfaces.^20,24^ The electrochemical response of the three cobalt catalysts immobilised onto ITO|*meso*ITO electrodes was compared to determine the loading of the Co catalysts to the metal oxide surface and the stability during voltammetry, specifically when cycling between the Co^III^, Co^II^ and Co^I^ oxidation states. The electrodes were prepared from ITO nanoparticles as described previously^24^ and were loaded with catalysts by immersing a cleaned slide into a 6 mM catalyst solution in dry DMF for 15 h. The ITO|*meso*ITO|**CoP^*n*^** electrodes were gently rinsed with fresh DMF, dried under N_2_ and studied in a **CoP^*n*^**-free DMF/TBABF_4_ electrolyte solution (0.1 M).

**Fig. 3:**
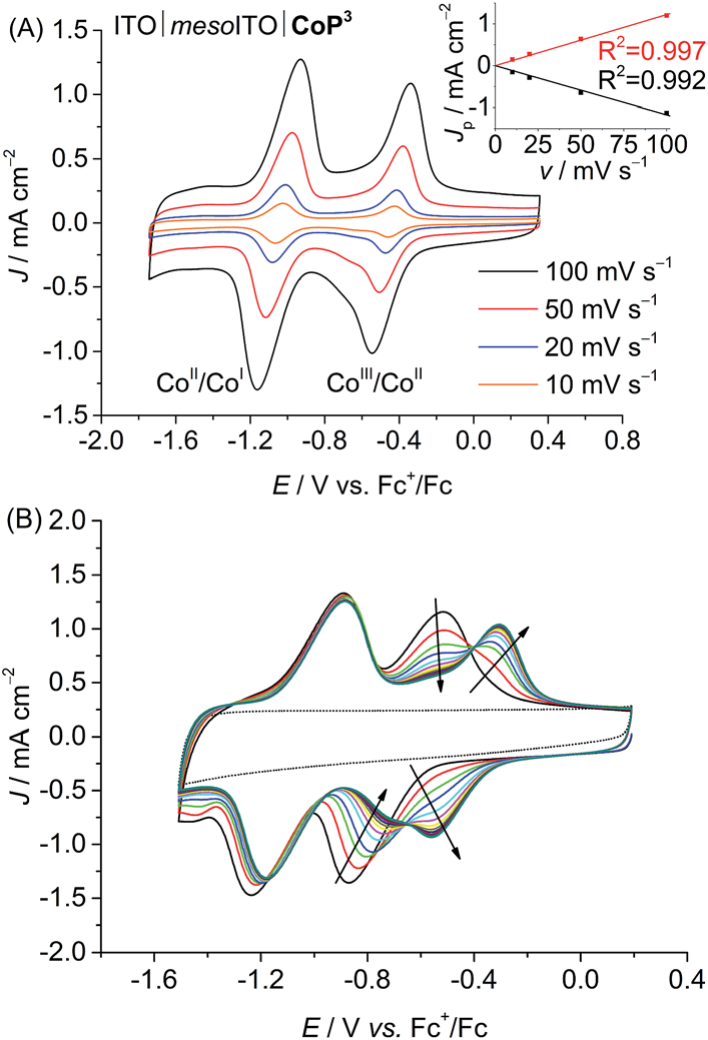
(A) CVs of ITO|*meso*ITO|**CoP^3^** in DMF/TBABF_4_ electrolyte (0.1 M) at different scan rates (10, 20, 50, 100 mV s^–1^). Inset: The correlation between the peak current density, *J*p (Co^II^/Co^I^), and scan rate, *v*, is shown. The black and red traces represent linear fits to the data points. (B) Consecutive CVs of ITO|*meso*ITO|**CoP^3^** in DMF/TBABF_4_ (0.1 M) at a scan rate of 100 mV s^–1^ showing cathodically induced replacement of the axial bromido ligand by DMF. The background of ITO|*meso*ITO without catalyst is shown as dotted line. Note that ligand exchange has already occurred in the CVs shown in (A).

CVs of the ITO|*meso*ITO|**CoP^3^** electrode in DMF/TBABF_4_ are shown in Fig. 3. A linear correlation between the peak current density, *J*_P_, of the reversible Co^II^/Co^I^ reduction at *E*_1/2_ = –1.03 V *vs.* Fc^+^/Fc and the scan rate, *v*, confirms that **CoP^3^** is immobilised on the ITO|*meso*ITO surface. The disappearance of the Co^III^/Co^II^ redox couple for the immobilised complex at *E*_1/2_ = –0.69 V *vs.* Fc^+^/Fc with the concomitant appearance of a new wave at *E*_1/2_ = –0.43 V *vs.* Fc^+^/Fc during consecutive scans is presumably due to a cathodically induced replacement of the axial bromido ligand by DMF.^16b,35^ CVs of ITO|*meso*ITO|**CoP^2^** show comparable features in DMF/TBABF_4_ (Fig. S22 and S23B†) with *E*_1/2_ = –0.59 V and –1.17 V *vs.* Fc^+^/Fc for Co^III^/Co^II^ and Co^II^/Co^I^, respectively. The determination of any *J*_P_–*v* correlation was not possible for ITO|*meso*ITO|**CoP^1^** due to the poor stability of the immobilised **CoP^1^** on ITO and subsequent rapid decrease of the redox waves within the first few scans (Fig. S23A;† see below).

The amounts of catalyst immobilised onto the mesoporous ITO electrodes were estimated by integration of the redox waves (reduction and oxidation) from the first CV scans in DMF/TBABF_4_ (Table 1). Loadings between 22 and 28 nmol cm^–2^ (referenced to the geometrical surface area of the electrode) were determined for the three ITO|*meso*ITO|**CoP^*n*^** electrodes. We only observed small differences in the loadings, which might be due to different spatial demands of the catalysts. Comparable results and trends were obtained when the integration of the redox waves was performed with CV scans recorded in aqueous electrolyte solution (Table S3, Fig. S24 and S25†) and loadings are comparable to a previously reported Ru-based compound on mesostructured ITO.^36^ The results show that **CoP^3^** binds well and with a comparable loading to **CoP^2^** to the metal oxide electrode despite only having one anchoring group.

**Table 1:** Loading of the three **CoP^*n*^** catalysts per geometrical surface area of ITO|*meso*ITO|**CoP^*n*^** electrodes as determined by integrating redox waves in CV traces recorded in DMF/TBABF_4_ at 100 mV s^–1^

Catalyst	*n* (**CoP^*n*^**)/nmol cm^–2^	
	First scan^*a*^	10^th^ scan^*b*^
**CoP^1^**	25.6 ± 1.1	5.6 ± 0.5
**CoP^2^**	28.1 ± 2.8	28.5 ± 3.6
**CoP^3^**	22.5 ± 1.5	22.7 ± 0.7

^*a*^Mean value with standard deviation (*σ*) for the first CV scan.

^*b*^Mean value with *σ* after 10 scans.

After 10 consecutive scans at *v* = 100 mV s^–1^ practically no desorption of **CoP^3^** and **CoP^2^** was observed, whereas approximately 80% of **CoP^1^** was lost from the ITO|*meso*ITO electrode (Table 1). As discussed above, reduction of low spin Co^III^ results in a labile Co^II^ and Co^I^ species, which leads to the loss of the Co(dmgH)_2_ core from the ITO-anchored phosphonated pyridine in **CoP^1^**.^7*b*^ This instability was not observed for **CoP^2^** and **CoP^3^**, demonstrating the much improved robustness when anchoring the cobalt catalysts with one (**CoP^3^**) or two (**CoP^2^**) phosphonic acid groups on the tetradendate equatorial (DO)(DOH)pn ligand to the ITO electrode (Fig. 3 and S23B†).^24^ **CoP^3^** therefore displays much higher stability on an electrode than **CoP^1^** and is significantly more active as a proton reduction catalyst than **CoP^2^** as shown by electrochemical investigations in solution.

### Photocatalytic studies

**Fig. 4:**
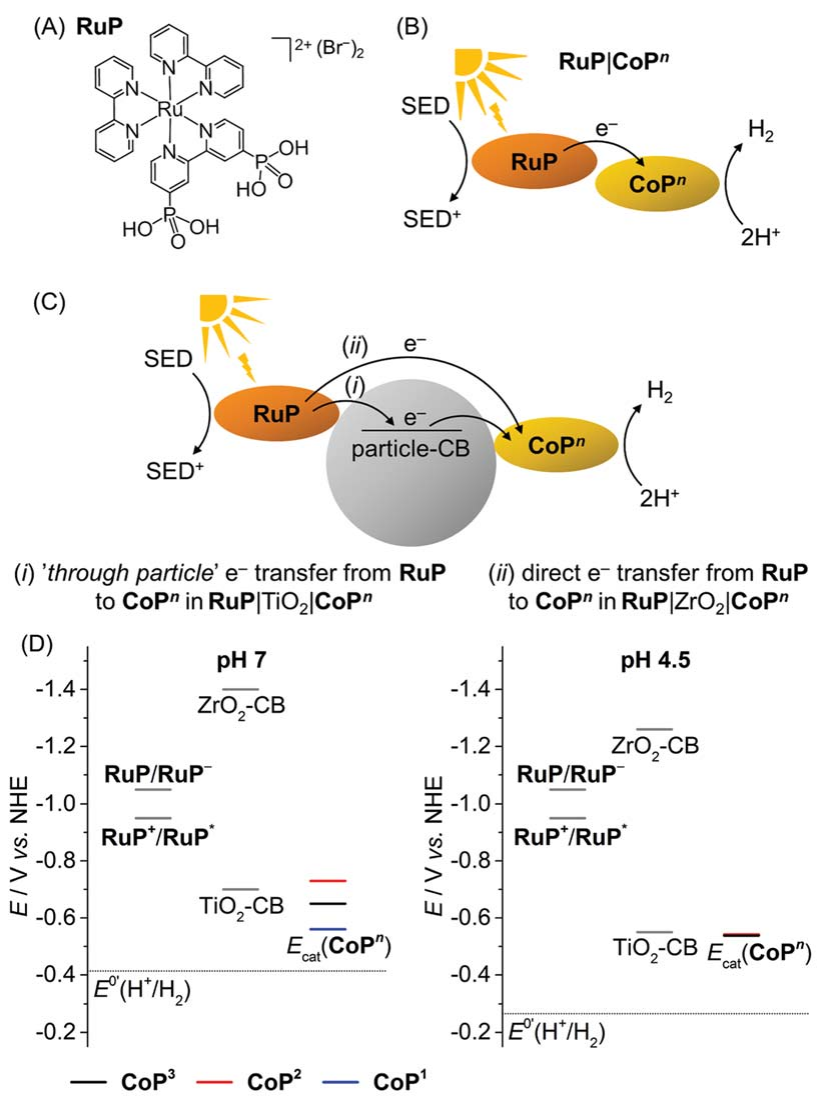
(A) Chemical structure of the photosensitiser **RuP**. (B and C) Electron transfer mechanisms from the photoexcited **RuP** dye to the catalyst **CoP^*n*^** in the homogenous and heterogeneous suspension systems with TiO_2_ and ZrO_2_ particles. The ‘*through particle*’ electron transfer pathway proceeds through oxidative quenching of **RuP** and is only accessible in **RuP**|TiO_2_|**CoP^*n*^** (see text). (D) Schematic energy diagram with the redox potentials of **RuP^+^**/RuP* and **RuP**/**RuP^–^** generated upon photoexcitation, conduction band potentials of the semiconductor particles (TiO_2_-CB and ZrO_2_-CB), the thermodynamic redox potential for proton reduction, *E*^0’^(H^+^/H_2_), and the catalytic proton reduction onset potentials, *E*_cat_, of the **CoP^*n*^** catalysts determined from CVs in TEOA/Na_2_SO_4_ (0.1 M each, pH 7) and acetate electrolyte solution (0.1 M, pH 4.5).

The photocatalytic activity of the **CoP^*n*^** catalysts was studied in solution and in heterogeneous suspension systems containing either TiO_2_ or ZrO_2_ nanoparticles with TEOA (0.1 M, pH 7) or AA (0.1 M, pH 4.5) as buffer and sacrificial electron donor (SED). [Ru^II^(2,2′-bipyridine)_2_(2,2′-bipyridine-4,4′-bisphosphonic acid)]Br_2_ (**RuP**, Fig. 4A) was used as photosensitiser. Photoexcited **RuP** (RuP*) can operate through an oxidative (*E*^0^(**RuP^+^**/RuP*) = –0.95 V *vs.* NHE)^37^ or reductive quenching mechanism (*E*^0^(RuP*/**RuP^–^**) = 1.07 V *vs.* NHE),^38^ which would generate **RuP^–^** (*E*^0^(**RuP**/**RuP^–^**) = –1.05 V *vs.* NHE).^9,38,39^ Photoinduced electron transfer from **RuP** to the **CoP^*n*^** catalyst can occur either directly (homogeneous system; Fig. 4B) or *via* the injection of electrons into the conduction band (CB) of the semiconductor TiO_2_ (*E*_CB_ = –0.70 V at pH 7; *E*_CB_ = –0.55 V *vs.* NHE at pH 4.5)^40^ by a ‘*through particle*’ mechanism (Fig. 4C).^9^ RuP* and **RuP^–^** are unable to transfer electrons into the more negative CB of ZrO_2_ (*E*_CB_ = –1.40 V *vs.* NHE at pH 7, *E*_CB_ = –1.26 V *vs.* NHE at pH 4.5),^41^ which only allows for direct electron transfer from photoexcited **RuP** to the catalyst as in the homogeneous system in **RuP**|ZrO_2_|**CoP^*n*^** (Fig. 4C). A comparison of the electrocatalytic onset potentials for proton reduction of the **CoP^*n*^** catalysts with the thermodynamic driving force from photogenerated RuP* and **RuP^–^**, and the semiconductors is summarised in Fig. 4D. It illustrates that photo-H_2_ evolution is thermodynamically possible with all three catalysts, but kinetic factors may have a detrimental effect on some of the systems.^42^

In a standard experiment, 0.1 μmol **CoP^*n*^** and 0.1 μmol **RuP** were used in 2.25 mL of aqueous solution containing the SED (homogeneous **RuP**|**CoP^*n*^** system) and 5 mg of metal oxide nanoparticles were added for the particle systems (**RuP**|TiO_2_|**CoP^*n*^** or **RuP**|ZrO_2_|**CoP^*n*^**). The samples were kept at 25 °C and irradiated with visible light from a solar light simulator equipped with an AM 1.5G, IR and UV filter (*λ* > 420 nm). The activity is expressed as Co-based turnover number, TON_Co_ (mol H_2_ per mol **CoP^*n*^**), which was obtained after four hours of visible light irradiation (Table 2). At this point, all systems had lost their photoactivity under these standard conditions.

**Table 2:** Results of visible light driven H_2_ evolution with **CoP^*n*^** and **RuP** in solution or in particle suspensions with TiO_2_ or ZrO_2_^*a*^

	TOF_Co_^*b*^ (1 h)/h^–1^	TON_Co_^*c*^ (4 h)	*n* ^*c*^ (H_2_)/μmol (4 h)
**pH 7 (TEOA)**			
**RuP**|**CoP^3^**	—	—	<0.03^*d*^
**RuP**|ZrO_2_|**CoP^3^**	—	—	<0.03^*d*^
**RuP**|TiO_2_|**CoP^3^**	10.3 ± 0.4	12.3 ± 0.3	1.23 ± 0.03
**RuP**|TiO_2_|**CoP^3^**_centr._^*e*^	*n.d.* ^*f*^	*n.d.* ^*f*^	0.74 ± 0.27
**RuP**|TiO_2_|**CoP^2^**	0.6 ± 0.1	2.4 ± 0.1	0.24 ± 0.01
**RuP**|TiO_2_|**CoP^1^**	44.0 ± 0.9	56.6 ± 2.2	5.66 ± 0.22
**RuP**|TiO_2_, no **CoP^3^**	*n.d.* ^*f*^	*n.d.* ^*f*^	0.14 ± 0.07

**pH 4.5 (AA)**			
**RuP**|**CoP^3^**	2.1 ± 0.6	3.1 ± 0.4	0.31 ± 0.04
**RuP**|ZrO_2_|**CoP^3^**	8.1 ± 2.2^*g*^	9.9 ± 0.2	0.99 ± 0.02
**RuP**|TiO_2_|**CoP^3^**	12.8 ± 0.6^*g*^	18.4 ± 0.5	1.84 ± 0.05
**RuP**|TiO_2_|**CoP^2^**	1.2 ± 0.2	1.2 ± 0.1	0.12 ± 0.01
**RuP**|TiO_2_|**CoP^1^**	—	—	<0.03^*d*^
**RuP**|TiO_2_, no **CoP^3^**	—	—	<0.03^*d*^
**RuP**|ZrO_2_, no **CoP^3^**	*n.d.* ^*f*^	*n.d.* ^*f*^	0.09 ± 0.02
**RuP**, no **CoP^3^**	—	—	<0.03^*d*^

^*a*^The following standard conditions were employed unless otherwise noted: AM 1.5G, 100 mW cm^–2^, *λ* > 420 nm irradiation, 0.1 μmol of **CoP^*n*^** and 0.1 μmol of **RuP** in homogenous solution or in suspensions with TiO_2_ or ZrO_2_ nanoparticles (5 mg) in aqueous TEOA or AA solution (2.25 mL, 0.1 M). Mean values ± standard deviation (*σ*) given from at least three different reaction vessels.

^*b*^TOF based on **CoP^*n*^** for the first hour of irradiation.

^*c*^TON based on **CoP^*n*^** and total of headspace H_2_ accumulated after four hours irradiation.

^*d*^Below the limit of detection by gas chromatography.

^*e*^Particles were loaded with the catalyst and the dye, centrifuged and re-suspended in fresh buffer solution prior to use.

^*f*^ *n.d.* = not defined (no **CoP^3^** present or amount of **CoP^3^** not precisely known).

^*g*^TOF is based on the maximum H_2_ evolution rate after the initial lag period.

**Fig. 5:**
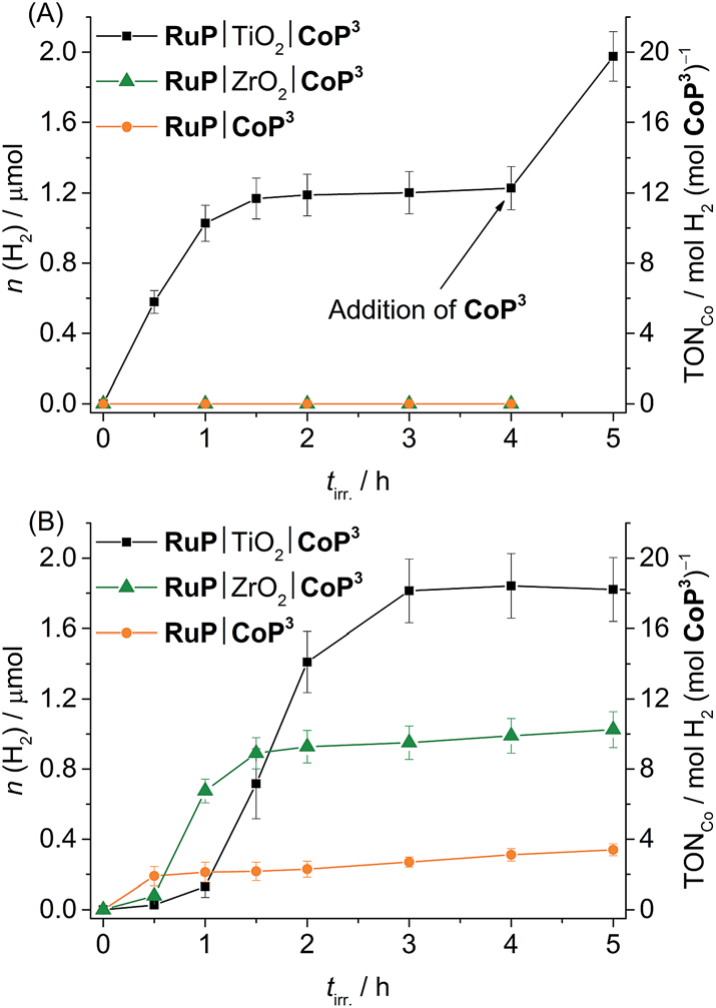
Photoactivity of **CoP^3^** expressed as total amount of headspace H_2_ over irradiation time and TON_Co_ (AM 1.5G, 100 mW cm^–2^, *λ* > 420 nm) in different systems (**RuP**|TiO_2_|**CoP^3^**, **RuP**|ZrO_2_|**CoP^3^** and **RuP**|**CoP^3^**) in (A) pH 7 TEOA (2.25 mL, 0.1 M) and (B) pH 4.5 AA solution (2.25 mL, 0.1 M). A 1 : 1 ratio of **CoP^3^** and **RuP** (0.1 μmol each) was used and either 5 mg of TiO_2_ or ZrO_2_ were added in case of particle systems.

We first investigated the photocatalytic activity of **CoP^3^** in pH 7 TEOA solution. No H_2_ was generated in the **RuP**|**CoP^3^** and **RuP**|ZrO_2_|**CoP^3^** systems, but **RuP**|TiO_2_|**CoP^3^** produced a TON_Co_ of 12.3 ± 0.3 (Fig. 5A). No H_2_ or only trace amounts of H_2_ were detectable when omitting **CoP^3^**, **RuP**, SED or light from this system or when CoBr_2_ was added instead of **CoP^3^** (Table S5†). Increasing the concentration of **CoP^3^** in **RuP**|TiO_2_|**CoP^3^** to 0.2 μmol resulted in a slight enhancement in the overall TON_Co_ (16.5 ± 0.5; Fig. S26A†). The highest TON_Co_ of 22.0 ± 1.5 was observed when the amount of **RuP** was increased to 0.2 μmol (Table S4 and Fig. S26B†).

The lack of photo-H_2_ evolution in the homogeneous and ZrO_2_-containing systems suggests that RuP* is not capable of reducing **CoP^3^** directly to initiate proton reduction which is in agreement with the previously reported inactivity of **RuP**|ZrO_2_|**CoP^1^**, **RuP**|**CoP^1^** and a [CoBr_2_((DO)(DOH)pn)] complex in combination with a Ru-dye and triethylamine as SED in solution.^20a,25b,39^ A possible explanation may be that the photoexcited state life-time of RuP* is too short-lived and the more reducing **RuP^–^** is not generated in aqueous TEOA solution.^43^ Addition of TiO_2_ facilitates oxidative quenching of RuP* and charge separation, which allows for efficient electron transfer from **RuP** to **CoP^3^***via* its CB in a ‘*through particle*’ mechanism, thereby triggering photoactivity of this system.^20a,39^ A comparable, surface-linker free cobalt diimine–dioxime catalyst with a pendant pyridine ligand was studied in solution using a Re photosensitiser and TEOA as sacrificial agent. A Co-based TON_Co_ of approximately 15 has been reported for this homogeneous photocatalytic system under near neutral conditions (pH 7.7).^25*c*^ The cobalt diimine–dioxime catalyst with a pendant pyridine ligand therefore keeps the full activity when immobilised on a semiconductor as is evident from the maximum TON_Co_ of 22.0 ± 1.5 observed with **RuP**|TiO_2_|**CoP^3^**.

Photo-H_2_ evolution activity of the deactivated **RuP**|TiO_2_|**CoP^3^** system was fully recovered by addition of fresh **CoP^3^** to the suspension (Fig. 5A), indicating complete degradation of **CoP^3^** within the first few hours of photocatalysis. To date, no detailed studies on possible degradation products of [Co(DO)(DOH)pn] catalysts are available, but partial regeneration of the catalyst by addition of fresh (DOH)_2_pn ligand to a deactivated system was reported, which suggests ligand degradation, most likely through hydrogenation.^25b,44^ The reduction of **CoP^3^** could also lead to a ligand radical species (Co^II^L˙, L = ligand) instead of the formal Co^I^ species.^24^ Reductive coupling of two Co^II^L˙ radical species might result in the formation of catalytically inactive dimer complexes.^45^ The formation of a Co-containing solid-state deposit would be another possible degradation pathway.^46^ The absence of photocatalytic activity after several hours of irradiation, the recovery of activity by addition of fresh **CoP^3^** and the lack of activity when replacing **CoP^3^** with CoBr_2_ support that a molecular Co species is the catalyst in the **RuP**|TiO_2_|**CoP^3^** system.

When stirring **CoP^3^** (0.1 μmol) with 5 mg TiO_2_ in an aqueous pH 7 TEOA solution, approximately 60% of the catalyst was attached to the particles as determined by spectrophotometry following *λ* = 259 nm (Fig. S27A†). **RuP** binds well to TiO_2_ and approximately 80% (*λ*_max_ = 288 and 455 nm) were adsorbed in the presence of 0.1 μmol **CoP^3^** (Fig. S27B†). The overlap of the strong absorption bands in **RuP** prevented the accurate determination of the **CoP^3^** loading in the presence of **RuP**. Approximately 60% of photocatalytic activity remained (0.74 ± 0.27 μmol H_2_) when unbound **CoP^3^** and **RuP** were removed from the pre-loaded particles by centrifugation and **RuP**|TiO_2_|**CoP^3^** was resuspended in a fresh TEOA buffer solution (Table 2). This observation agrees well with the loading of **CoP^3^** and shows that the majority of attached **CoP^3^** remained on the particle surface and was not replaced by the dye (5 mg P25 TiO_2_ nanoparticles have a loading capacity of approximately 0.25 μmol **RuP**).^6*d*^

Full spectrum irradiation (AM 1.5G, 100 mW cm^–2^, no UV filter) of dye-free TiO_2_|**CoP^3^** resulted in a TON_Co_ of 17.2 ± 1.3 in pH 7 TEOA solution, demonstrating that conduction band electrons can be transferred to **CoP^3^**. The photo-H_2_ production activity decreased by 97% when phosphate buffer (50 mM, pH 7) was added to the system (Fig. S28†). The phosphate anions and the phosphonic acid group in **CoP^3^** compete for surface binding sites on TiO_2_. This experiment demonstrates that binding of **CoP^3^** to the TiO_2_ nanoparticle *via* the (–PO_3_H_2_) anchoring group is essential for effective electron transfer from the TiO_2_ conduction band to the catalyst^20*a*^ and further supports that a molecular catalyst rather than a solid state deposit is active on TiO_2_.

Finally, an unoptimised external quantum efficiency (EQE) of 0.35 ± 0.02% was determined for the **RuP**|TiO_2_|**CoP^3^** system (0.1 μmol **RuP**, 5 mg TiO_2_, 0.2 μmol **CoP^3^**) in an aqueous pH 7 TEOA solution (0.1 M) after 1 h irradiation at *λ* = 465 nm (*I* = 22 mW cm^–2^), which is close to the absorption maximum of **RuP** (*λ*_max_ = 455 nm). This value is comparable to the previously reported EQE for **RuP**|TiO_2_|**CoP^1^** (1.0 ± 0.2%)^39^ and colloidal systems containing carbon nitrides and molecular Ni catalysts (0.37 and 1.51%).^12,47^

In pH 4.5 AA solution, a TON_Co_ of 18.4 ± 0.5, 9.9 ± 0.2 and 3.1 ± 0.4 was observed with **RuP**|TiO_2_|**CoP^3^**, **RuP**|ZrO_2_|**CoP^3^** and **RuP**|**CoP^3^**, respectively (Table 2 and Fig. 5B). The three systems were completely deactivated after 4 h of visible light irradiation. Control experiments with CoBr_2_ instead of **CoP^3^** and in the absence of **CoP^3^**, **RuP**, electron donor or light showed no or only trace amounts of H_2_ (Table S8†). The different activity of the three systems can be explained by two different mechanisms occurring under these experimental conditions (pH 4.5, AA). Previous studies have shown that RuP* is readily quenched oxidatively on TiO_2_ by electron transfer to the TiO_2_ conduction band in the picosecond time-scale,^9,48^ whereas RuP* undergoes reductive quenching by AA to generate **RuP^–^** in solution or in the ZrO_2_ system.^9^ Inefficient photocatalytic H_2_ evolution has been previously reported for [CoX_2_(DO)(DOH)pn] complexes in combination with a Ru-dye in AA.^49^ The oxidative quenching pathway in the TiO_2_-containing system provides a possible explanation for the improved photocatalytic activity of **RuP**|TiO_2_|**CoP^3^**.

The initial lag period of photo-H_2_ evolution in AA was dependent on the ratio of **CoP^3^** to **RuP** and is presumably due to the slow accumulation of Co^I^ species, which is required to enter the catalytic cycle. An increased lag phase with enhanced photostability and a higher final TON_Co_ was observed in all three photocatalytic systems when changing the **CoP^3^** : **RuP** ratio from 1 : 1 to 2 : 1. At a **CoP^3^** : **RuP** ratio of 1 : 2, a reduced lag phase with a shorter lifetime of photocatalysis and a somewhat lower final TON_Co_ was achieved (Table S7 and Fig. S29†). Recovery of the photocatalytic activity of **RuP**|TiO_2_|**CoP^3^** by addition of either fresh **CoP^3^** or **RuP** was not successful suggesting simultaneous degradation of both, dye and catalyst. By providing new **CoP^3^** and **RuP**, the initial photocatalytic activity of the **RuP**|TiO_2_|**CoP^3^** system could be regained (Fig. S30†). Photo-degradation of **RuP** in AA has been observed previously.^9^ Similar pathways as discussed above might account for degradation of the Co catalyst in an aqueous AA solution.

Finally, the photocatalytic activity of the colloidal **RuP**|TiO_2_|**CoP^3^** system was compared to the activity of **CoP^1^** and **CoP^2^** using standard conditions (0.1 μmol **CoP^*n*^** and 0.1 μmol **RuP** on 5 mg TiO_2_). In TEOA solution (0.1 M, pH 7), a TON_Co_ of 56.6 ± 2.2 was obtained for **CoP^1^**,^20*b*^ whereas the **RuP**|TiO_2_|**CoP^2^** system only produced small amounts of H_2_ (TON_Co_ = 2.4 ± 0.1; Table 2 and Fig. S31A†). In AA at pH 4.5, only traces of H_2_ were produced with **CoP^1^**, which is catalytically unstable under acidic conditions (see above). A TON_Co_ of approximately 1 was achieved for **CoP^2^** during 4 h visible light irradiation in AA (Table 2, Fig. S31B†).

The results from photocatalytic experiments are in agreement with trends observed during electrochemical investigation of the three catalysts: **CoP^1^** shows the fastest turnover rate at neutral pH, whereas **CoP^3^** is the most active catalyst in an aqueous acidic solution. However, **CoP^3^** is the best and most suitable catalyst when activity *and* stability on the metal oxide surface are taken into account. **CoP^2^** displays strong attachment to metal oxides, but it shows overall modest catalytic activity. **CoP^1^** is not stable during turnover in a pH 4.5 AA solution and can therefore not act as a catalyst under acidic conditions. The high photoactivity of **CoP^1^** at pH 7 despite its labile anchoring to **RuP**|TiO_2_ particles in the colloidal suspension can be explained as follows: the Co(dmgH)_2_ core of **CoP^1^** is released during catalysis but can re-coordinate to a TiO_2_-anchored pyridine ligand by a ‘hop-on, hop-off’ mechanism through a high probability of collision in the bulk of the suspension. When **CoP^1^** is immobilised on an electrode such as ITO|*meso*ITO, however, the Co(dmgH)_2_ core will be released from the surface and will diffuse into the bulk solution, where it will not readily diffuse back to the electrode surface.

## Conclusions

In summary, a new cobalt diimine–dioxime H_2_ evolution catalyst (**CoP^3^**) is described that features a stable binding site for attachment to metal oxide surfaces and a pendant pyridine ligand to enhance the catalytic activity. **CoP^3^** was prepared in six steps and characterised by NMR, UV-vis and ATR-IR spectroscopy, mass spectrometry and elemental analysis. Electrochemical investigation of the new catalyst revealed that it is electrocatalytically active for proton reduction in aqueous solution over a wide pH range. **CoP^3^** attaches with high loading and good stability to a mesostructured Sn-doped In_2_O_3_ electrode. We demonstrate that **CoP^3^** produces H_2_ photocatalytically in dye-sensitised systems under visible light irradiation at neutral and acidic pH with different sacrificial reagents and showed that H_2_ evolution is improved in the presence of TiO_2_ particles compared to homogeneous systems. **CoP^3^** displays significant advantages over previously reported immobilised Co catalysts as it shows a higher catalytic proton reduction activity *and* provides a strong and more stable anchoring to metal oxides surfaces on electrodes.

Overall, our work emphasises the necessity for elaborated molecular catalyst design with regard to the assembly of efficient metal oxides–molecular catalyst hybrids and their application in (photo-)electrochemical cells. The availability of thorough experimental and theoretical studies for cobaloxime and cobalt diimine–dioxime catalysts enabled us to rationally design a catalyst with improved activity and stability on electrodes.

## Supplementary Material

Supplementary information
