# Supplementary material for: Enhancing H2 evolution performance of an immobilised cobalt catalyst by rational ligand design
Source: Chem Sci. 2015 Feb 2;6(5):2727–36. doi: 10.1039/c4sc03946g (PMC5654411; doi:10.1039/c4sc03946g)
Supplement: Supplementary file 1 [file SC-006-C4SC03946G-s001.pdf]

Electronic Supplementary Material (ESI) for Chemical Science.
This journal is © The Royal Society of Chemistry 2015

Electronic Supporting Information

Enhancing H_2 Evolution Performance of an Immobilised Cobalt Catalyst by Rational Ligand Design

Janina Willkomm, Nicoleta M. Muresan, Erwin Reisner*

Christian Doppler Laboratory for Sustainable SynGas Chemistry, Department of Chemistry, University of Cambridge, Lensfield Road, Cambridge CB2 1EW, UK

**Corresponding author: reisner@ch.cam.ac.uk*

Contents

Experimental Section

Supporting Tables S1–S8

Supporting Figures S1–S32

Supporting References

Experimental Section

Materials and Methods

All chemicals were purchased from commercial suppliers and used without further purification unless otherwise noted. Chemicals for analytical measurements were of the highest available purity. *Aeroxide* TiO_2 P25 particles (anatase/rutile: 8/2 mixture, average particle size = 21 nm) were a gift from *Evonik Industries* and ZrO_2 nanoparticles (99.9%, 20−30 nm) were obtained from *Skyspring Nanomaterials Inc.* **CoP^1**, **CoP^2** and [Ru(2,2'-bipyridine)$_2$(2,2'-bipyridine-4,4'-bisphosphonic acid)]Br_2 (**RuP**) were synthesised according to literature procedures.[1] Air and moisture sensitive reactions were carried out using Schlenk technique or in a *MBraun UniLab* glovebox. Freshly dried and distilled solvents were used for moisture-sensitive experiments and compounds.

Preparation of Buffer Solutions

Acetate buffer (0.1 M, pH 4.5) was prepared by mixing 0.1 M acetic acid and 0.1 M sodium acetate solutions in a specific ratio to obtain the desired pH. Ascorbic acid (AA) solutions (0.1 M, pH 4.5) were freshly prepared before each experiment and triethanolamine·HCl (TEOA·HCl) was used to prepare TEOA solutions (0.1 M, pH 7). The pH of each buffer was adjusted with a NaOH solution (1.0 M). The final pH was confirmed after diluting to the final concentration of 0.1 M. The Britton-Robinson buffer for pH dependency studies contains H_3BO_3, H_3PO_4, CH_3COOH (0.04 M each) and the pH was adjusted using a NaOH solution (0.2 M).

Physical Measurements

^{1}H, ^{13}C, ^{31}P and NOE NMR spectra were recorded on a *Bruker* 400 MHz NMR spectrometer at room temperature (r.t.). The measurements were carried out in commercially available deuterated solvents. The residual solvent peak was used as an internal standard in ^{1}H and ^{13}C

NMR spectra. H_3PO_4 (85%) was used as an external reference for ^{31}P NMR spectra. For the 1D NOESY spectrum, the ^{1}H signal at 19.2 ppm was pre-irradiated until saturation and the response was recorded. High resolution (HR)-mass spectra were recorded in methanol using a *ThermoScientific Orbitrap Classic* mass spectrometer. Elemental analysis was carried out by the University of Cambridge Microanalysis Service using a *Perkin-Elmer 240* Elemental Analyser. Infrared spectra were recorded on a *Thermo Scientific Nicolet iS50* FT-IR spectrometer using ATR. UV-vis spectra were collected using a *Varian Cary 50 Bio* UV-vis spectrometer.

Electrochemistry

Cyclic voltammetry was carried out using an *EmStat 3+* potentiostat, *IviumStat* or *Ivium CompactStat* potentiostat/galvanostat. Measurements were performed at 25 °C with water-jacketed electrochemical cell connected to a water circulator under N_2 in a three electrode configuration. All electrolyte solutions were purged with N_2 for 10 min prior to the measurements to remove O_2. A glassy carbon disk (3 mm diameter) or ITO|*meso*ITO (geometrical area = 0.25 cm^2) working and a Pt mesh counter electrode were employed. In water, a Ag/AgCl/KCl_{sat} reference electrode was used and the potentials were converted to the normal hydrogen electrode (NHE) by addition of +0.197 V.[2] Measurements in DMF/$TBABF_4$ electrolyte solution ($TBABF_4$ = tetrabutylammonium tetrafluoroborate, 0.1 M) were performed using a Ag/AgCl reference electrode. Ferrocene was added as an internal standard and the redox potentials were referenced against the Fc^+/Fc redox couple. The catalyst concentration was 0.8 mM when studied in solution. Co^{III}/Co^{II} and Co^{II}/Co^{I} redox potentials are given as $E_{1/2}$ and were determined from the cathodic and anodic peak potentials (for reversible redox couples) and as half peak potential for irreversible redox waves.

Immobilisation of Molecular Catalysts on ITO|mesoITO Electrodes

ITO|*meso*ITO electrodes were prepared as previously reported.[1a] 2 μL of an ITO nanoparticle suspension (*Sigma Aldrich*, diameter < 40 nm, BET = 27 m^2 g^{-1}, 90% In_2O_3, 20wt% in 5 M acetic acid in ethanol) were drop-casted onto ITO-coated glass slides (*Vision Tek System Ltd.* 30 Ω sq^{-1}, 1 x 2 cm^2) with *Scotch*® tape (3M) as spacers (0.5 x 0.5 cm^2). After drying in air, the slides were annealed at 350 ºC for 20 min (heating rate to 350 ºC: 4 ºC min^{-1}). The geometrical surface area of the mesoporous ITO coating was 0.25 cm^2 (thickness: 13 μm)[1a] and the electrodes were cleaned with 2-propanol and acetone and dried under a stream of N_2 prior use. The ITO|*meso*ITO electrodes were then immersed into a 6 mM solution of the catalyst (**CoP^1**, **CoP^2** or **CoP^3**) in dry DMF for 15 h to allow for adsorption of the catalyst. The hybrid electrodes were gently rinsed with dry DMF and dried under a stream of N_2.

Photocatalytic Experiments

Photocatalytic experiments were performed using a *Newport Oriel* solar light simulator (100 mW cm^{-2}, AM 1.5G). The light source was equipped with a water filter to remove IR irradiation and a 420 nm cut-off filter to eliminate UV irradiation if required. Samples were prepared by sonicating 5 mg of nanoparticles (TiO_2 or ZrO_2) in an appropriate volume of buffered solution (AA, 0.1 M, pH 4.5 or TEOA buffer, 0.1 M, pH 7) for 10 min followed by addition of the catalyst (**CoP^1**, **CoP^2** or **CoP^3**, 1 mM in water). After stirring the resulting suspension for 10 min, the **RuP** dye (1 mM in water) was added, the sample vial sealed and purged with N_2 containing 2% CH_4 as internal GC standard for 10 min. Solution samples were prepared as described above without addition of any particles. The total volume of the suspension/solution was 2.25 mL and the temperature of the photoreactor was kept at 25 ºC with a water-jacketed and temperature-controlled water bath during the experiment. The remaining headspace (5.59 mL) of the photoreactor was analysed by gas chromatography

(GC, *Agilent* 7890A Series) in regular time intervals. The GC was equipped with a 5 Å molecular sieve column (45 °C) and a thermal conductivity detector. N_2 was used as carrier gas (flow rate: 3 mL min^{-1}). All experiments were repeated at least three times. The mean value and standard deviation σ were calculated. A minimum σ of 10 % was assumed.

Quantification of Attachment of CoP^3 and RuP to TiO_2 Nanoparticles

TiO_2 nanoparticles (5 mg) were sonicated in 2.15 mL of TEOA buffer for 10 min, followed by addition of **CoP^3** (0.1 µmol, 1 mM in water). After stirring the mixture for 10 min, the suspension was centrifuged (8000 rpm, 10 min), the supernatant separated from the nanoparticles and centrifuged again (8000 rpm, 10 min). The UV-vis spectrum of the supernatant was recorded and compared to the UV-vis spectrum recorded prior to addition of TiO_2. The loading of **RuP** onto TiO_2 particles was studied in the presence of **CoP^3** (0.1 µmol each). Samples were prepared as described above and the suspension was stirred for further 10 min after the addition of **RuP** (0.1 µmol, 1 mM in water). If the centrifuged particles were used in photocatalysis, they were re-dispersed in 2.25 mL of a fresh TEOA buffer.

Quantum efficiency measurement

The external quantum efficiency (EQE) was determined for **RuP**|TiO_2|**CoP^3** in pH 7 TEOA solution and the sample was prepared following the standard procedure as described above. The photoreactor was purged with N_2 (2% CH_4 as internal standard) for 10 min, followed by irradiation with blue light (λ = 465 nm) from an *Ivium modulight* LED light source. The light intensity was I = 22 mW cm^{-2} and the irradiated area was A = 3.6 cm^2. The headspace gas in the reactor was analysed by GC. The following equation was used to determine the EQE from the amount of H_2 produced after 1 h irradiation:

$$EQE(\%) = \frac{n(\mathrm{H_2}) \cdot \mathrm{N_A} \cdot \mathrm{h} \cdot \mathrm{c} \cdot 2}{\lambda \cdot t \cdot I \cdot A} \cdot 100$$

where $n(H_2)$ = moles of H_2 produced, N_A = Avogadro constant, h = Planck constant; c = speed of light and t = irradiation time. Note that the obtained EQE is a lower limit of quantum efficiency of the system, as it was assumed that all incident light was absorbed by the suspension.

Synthesis and Characterisation of Molecular Compounds

*2-(4-Bromobenzyl)malononitrile (**1**)* was prepared according to the literature with slight modifications.[3] 4-Bromobenzaldehyde (1.3 g, 20 mmol) was added to a solution of malononitrile (3.7 g, 20 mmol) in water/ethanol (50 mL, 95/5) and the mixture was stirred for 2 h at r.t. to form the intermediate condensation product. $NaBH_4$ (200 mg, 5 mmol) was slowly added to the suspension until a clear solution was obtained. After stirring for 1 h, water (200 mL) was added to precipitate the product, which was filtered off, washed with water and dried under high vacuum at r.t. to give **1** as an off-white solid (Yield: 3.7 g, 80%). ^{1}H NMR (400 MHz, $CDCl_3$): δ/ppm = 7.58 (d, ^3J(H,H)=8.5 Hz, 2H, Ph), 7.25 (d, ^3J(H,H)=8.5 Hz, 2H, Ph), 3.94 (t, ^3J(H,H)=6.7 Hz, 1H, CH), 3.29 (d, ^3J(H,H)=6.7 Hz, 2H, CH_2); HR-ESI-MS, m/z (MeOH), +ve: calculated for $[M+H]^+$ 232.9720, found 232.9723 (100%); ATR-IR: 2260 cm^{-1} (CN, medium).

*Compound **2**.* Compound **1** (2.0 g, 8.5 mmol), PPh_3 (2.0 g, 7.6 mmol) and $Pd(PPh_3)_4$ (0.4 g, 4 mol%) were dissolved in dry tetrahydrofuran (40 mL). Triethylamine (1.4 mL, 10.1 mmol) and diethyl phosphite (1.4 mL, 11.7 mmol) were added and the reaction mixture was refluxed under N_2 for 48 h. After cooling to r.t., the reaction mixture was filtered and concentrated to dryness under reduced pressure. The crude product was purified by column chromatography (silica, chloroform followed by a chloroform/methanol gradient of 0 to 3% methanol). *Compound **2*** was isolated as a pale yellow oil (Yield: 1.8 g, 73%). ^{1}H NMR (400 MHz, $CDCl_3$): δ/ppm = 7.89 (dd, ^3J(H,H)=8.5 Hz, ^3J(H,P)=13.2 Hz, 2H, Ph), 7.48 (dd, ^4J(H,P)=3.8

Hz, ^3J(H,H)=8.5 Hz, 2H, Ph), 4.26-4.07 (m, 4H, POCH_2CH$_3$), 4.01 (t, ^3J(H,H)=6.7 Hz, 1H, CH), 3.37 (d, ^3J(H,H)=7.0 Hz, 2H, CH$_2$), 1.37 (td, ^4J(H,P)=0.6 Hz, ^3J(H,H)=7.0 Hz, 6H, POCH$_2$CH_3); ^{13}C NMR (101 MHz, CDCl$_3$): δ/ppm = 137.6 (d, ^4J(C,P)=3.2 Hz, C, Ph), 133.1 (d, ^3J(C,P)=9.6 Hz, CH, Ph), 129.7 (d, ^2J(C,P)=15.2 Hz, CH, Ph), 129.7 (d, ^1J(C,P)=189.4 Hz, C, Ph), 112.3 (s, CN), 62.7 (d, ^2J(C,P)=5.6 Hz, POCH$_2$CH$_3$), 36.9 (s, CH$_2$) 24.9 (s, CH) 16.7 (d, ^3J(C,P)=6.4 Hz, POCH$_2$$CH_3$); ^{31}P NMR (162 MHz, CDCl$_3$): δ/ppm = 18.7 (s); HR-ESI-MS, m/z (MeOH), +ve: calculated for $[M+H]^+$ 293.1050, found 293.1043 (100%); ATR-IR: 2260 cm^{-1} (CN, weak), 1235 cm^{-1} (P=O, strong), 1020 cm^{-1} (P–OR, strong).

Compound 3. Compound **2** (0.55 g, 1.88 mmol) was dissolved in dry acetone (10 mL). 2-(Bromomethyl)pyridine·HBr (0.48 g, 1.88 mmol) and K_2CO_3 (0.57 g, 4.13 mmol) were added and the mixture was stirred under N_2 at r.t. for 3 days. The reaction mixture was filtered through a pad of silica and the filtrate was concentrated to approximately 2 mL under reduced pressure. Addition of dry diethyl ether to the oily residue resulted in precipitation of the product as a pale orange solid, which was filtered off, washed with diethyl ether and dried under high vacuum at r.t. (Yield: 0.42 g, 58%). ^{1}H NMR (400 MHz, CDCl$_3$): δ/ppm = 8.71 (dd, ^4J(H,H)=2.1 Hz, ^3J(H,H)=4.7 Hz, 1H, py), 7.88 (dd, ^3J(H,H)=8.5 Hz, ^3J(H,P)=13.2 Hz, 2H, Ph), 7.79 (td, ^4J(H,H)=1.8, ^3J(H,H)=7.7 Hz, 1H, py), 7.59 (dd, ^4J(H,P)=3.5 Hz, ^3J(H,H)=8.5 Hz, 2H, Ph), 7.41 (t, ^3J(H,H)=7.9 Hz, 1H, py), 7.36 (dd, ^3J(H,H)=4.9 Hz, ^3J(H,H)=7.7 Hz, 1H, py), 4.26-4.08 (m, 4H, POCH_2CH$_3$), 3.49 (s, 2H, CH$_2$), 3.47 (s, 2H, CH$_2$), 1.37 (td, ^4J(H,P)=0.6 Hz, ^3J(H,H)=7.0 Hz, 6H, CH$_3$); ^{13}C NMR (101 MHz, CDCl$_3$): δ/ppm = 152.9 (s, C, py), 150.1 (s, CH, py), 137.5 (s, CH, py), 137.0 (d, ^4J(C,P)=3.2 Hz, C, Ph), 132.6 (d, ^3J(C,P)=10.4 Hz, CH, Ph), 131.0 (d, ^2J(C,P)=15.2 Hz, CH, Ph), 129.5 (d, ^1J(C,P)=189.3 Hz, C, Ph), 124.9 (s, CH, py), 123.8 (s, CH, py), 115.2 (s, CN), 62.6 (d, ^2J(C,P)=5.6 Hz, POCH$_2$CH$_3$), 44.2 (s, CH$_2$), 42.8 (s, CH$_2$), 38.9 (s, C(CN)$_2$), 16.7 (d, ^3J(C,P)=6.4 Hz, POCH$_2$$CH_3$); ^{31}P NMR (162 MHz, CDCl$_3$): δ/ppm = 19.0; HR-ESI-MS, m/z

(MeOH), +ve: calculated for $[M+H]^+$ 384.1472, found 384.1464 (100%); Elemental analysis calculated for $C_{20}H_{22}N_3O_3P$: C 62.66%, H 5.78%, N 10.96%, P 8.08%; found: C 62.43%, H 5.77%, N 10.95%, P 7.86%; ATR-IR: 2250 cm^{-1} (CN, weak), 1240 cm^{-1} (P=O, strong), 1020 cm^{-1} (P–OR, strong).

Compound 4. Borane·tetrahydrofuran (3.6 mL, 3.6 mmol) was slowly added to a solution of compound **3** (0.3 g, 0.77 mmol) in dry tetrahydrofuran (9 mL). The mixture was stirred under N_2 at r.t. for 24 h. Methanol (20 mL) was added slowly to quench excess borane and the solvent was removed under vacuum. The residue was dissolved in dichloromethane and concentrated again under reduced pressure. Compound **4** was obtained as a viscous oil (Yield: 0.30 g, 99%). The product was used for the next step without further purification. ^1H NMR (400 MHz, $CDCl_3$): δ/ppm = 8.57 (dd, ^4J(H,H)=1.9 Hz, ^3J(H,H)=4.8 Hz, 1H, py), 7.75 (dd, ^3J(H,H)=8.2 Hz, ^3J(H,P)=13.2 Hz, 2H, Ph), 7.61 (dt, ^4J(H,H)=1.9 Hz, ^3J(H,H)=7.7 Hz, 1H, py), 7.42 (dd, ^4J(H,P)=4.1 Hz, ^3J(H,H)=8.5 Hz, 2H, Ph), 7.20-7.13 (m, 2H, py), 4.24-4.05 (m, 4H, $POC\mathit{H_2}CH_3$), 2.85 (s, 2H, CH_2), 2.83 (s, 2H, CH_2), 2.52-2.38 (m, 4H, $C\mathit{H_2}NH_2$), 1.36 (dt, ^4J(H,P)=0.6 Hz, ^3J(H,H)=7.0 Hz, 6H, $POCH_2C\mathit{H_3}$); ^{13}C NMR (101 MHz, $CDCl_3$): δ/ppm = 159.2 (s, C, py), 149.1 (s, CH, py), 135.9 (s, CH, py), 131.5 (d, ^3J(C,P)=10.4 Hz, CH, Ph), 130.7 (d, ^2J(C,P)=14.7 Hz, CH, Ph), 124.8 (s, CH, py), 121.2 (s, CH, py), 62.0 (d, ^2J(C,P)=5.2 Hz, $PO\mathit{C}H_2CH_3$), 45.2 (s, CH_2), 45.0 (s, $\mathit{C}(CH_2NH_2)$, 41.5 (s, CH_2), 39.8 (s, CH_2), 16.3 (d, ^3J(C,P)=6.1 Hz, $POCH_2\mathit{C}H_3$); ^{31}P NMR (162 MHz, $CDCl_3$): δ/ppm = 19.3; HR-ESI-MS, m/z (MeOH), +ve: calculated for $[M+H]^+$ 392.2098, found 392.2098 (100 %); ATR-IR: 3375, 3300 cm^{-1} (N–H, medium), 1570 cm^{-1} (N–H, medium), 1235 cm^{-1} (P=O, medium), 1015 cm^{-1} (P–OR, strong).

*[Co^{III}Br((DO)(DOH)(4-BnPO_3Et_2)(2-CH_2py)pn)]Br (**$^{Et}CoP^3$**).* 2,3-Butanedione monoxime (303 mg, 3.1 mmol) was added to a solution of compound **4** (300 mg, 0.78 mmol) in dry

methanol (7.5 mL) and the solution was stirred under N_2 at r.t. for 5 days. A solution of $CoBr_2 \cdot 6H_2O$ (127 mg, 0.39 mmol) in methanol (3 mL) was added, which resulted in the formation of a dark red solution. Exposure of the solution to air for 5 min resulted in a color change to dark red-brown, indicating the oxidation of Co^{II} to Co^{III}. The solvent was evaporated under reduced pressure and the oily residue was washed with diethyl ether. The precipitate was filtered off and washed with diethyl ether. Purification by column chromatography (Sephadex LH20, methanol) afforded the complex as dark red-brown solid (Yield: 270 mg, 45%). ^{1}H NMR (400 MHz, $CDCl_3$): δ/ppm = 19.09 (s, 1H, OHO), 7.86-7.66 (m, 5H, Ph, py), 7.55 (td, ^{4}J(H,H)=1.4 Hz, ^{3}J(H,H)=7.8 Hz, 1H, py), 7.28-7.22 (m, 1H, py), 6.98 (td, ^{4}J(H,H)=1.4 Hz, ^{3}J(H,H)=6.8 Hz, 1H, py), 4.96 (d, ^{1}J(H,H)=15.0 Hz, 2H, CH_2), 4.21-3.99 (m, 8H, CH_2, $POC\mathit{H_2}CH_3$), 3.67 (s, 2H, CH_2), 2.73 (s, 3H, CH_3), 2.72 (s, 3H, CH_3) 2.45 (s, 6H, CH_3), 1.33 (t, ^{3}J(H,H)=7.2 Hz, 6H, $POCH_2C\mathit{H_3}$); ^{13}C NMR (101 MHz, $CDCl_3$): δ/ppm = 177.2 (s, CN), 164.9 (s, C, py), 158.5 (s, CN), 149.0 (s, CH, py), 140.2 (d, ^{4}J(C,P)=2.5 Hz, C, Ph), 139.2 (s, CH, py), 132.0 (d, ^{3}J(C,P)=10.8 Hz, CH, Ph), 131.6 (d, ^{2}J(C,P)=15.8 Hz, CH, Ph), 130.8 (s, CH, py), 127.6 (d, ^{1}J(C,P)=190.7 Hz, C, Ph), 123.3 (s, CH, py), 62.3 (d, ^{2}J(C,P)=5.8 Hz, $PO\mathit{C}H_2CH_3$), 59.2 (s, $C\mathit{C}H_2N$), 44.8 (s, CH_2), 43.8 (s, CH_2), 42.1 (s, $\mathit{C}(CH_2N)_2$), 19.1 (s, CH_3), 16.4 (d, ^{3}J(C,P)=6.6 Hz, $POCH_2\mathit{C}H_3$), 14.0 (s, CH_3); ^{31}P NMR (162 MHz, $CDCl_3$): δ/ppm = 19.0 (s); HR-ESI-MS, m/z (MeOH), +ve: calculated for $[M]^+$ 694.1199, found 694.1200 (100%); Elemental analysis calculated for $C_{28}H_{39}Br_2CoN_5O_5P$: C 43.37%, H 5.07%, N 9.03%, P 3.99% Br 20.61%; found: C 43.30%, H 5.63%, N 8.77%, P 4.15%, Br 20.56%; ATR-IR: 1240 cm^{-1} (P=O, medium), 1015 cm^{-1} (P–OR, medium).

$[Co^{III}Br((DO)(DOH)(4\text{-}BnPO_3H_2)(2\text{-}CH_2py)pn)]Br$ ***(CoP^3)***. A solution of bromotrimethyl-silane (91.9 mg, 0.6 mmol) in dry dichloromethane (5 mL) was added to a solution of **^{Et}CoP^3** (120 mg, 0.15 mmol) in dry dichloromethane (10 mL). The mixture was stirred under N_2 at

r.t. for 42 h. The solvent was evaporated and the residue dissolved in methanol (20 mL). The red-brown solution was stirred for 1 h at r.t. and then concentrated to dryness under reduced pressure. The residue was washed with dichloromethane and diethyl ether and was purified by column chromatography (Sephadex LH20, methanol). **CoP^3** was obtained as a dark red-brown solid (Yield: 70 mg, 65%). ^{1}H NMR (400 MHz, DMSO-d_6) δ/ppm = 19.17 (s, 1H, OHO), 7.62-7.80 (m, 6H, ph, py), 7.35 (d, ^3J(H,H)=8.2 Hz, 1H, py), 7.26 (t, ^3J(H,H)=6.8 Hz, 1H, py), 4.52 (d, ^2J(H,H)=15.0 Hz, 2H, CH_2), 3.91 (d, ^2J(H,H)=15.3 Hz, 2H, CH_2), 3.44 (s, 2H, CH_2), 3.20 (s, 2 H, CH_2), 2.61 (s, 2x3H, CH_3), 2.41 (s, 6H, CH_3); ^{13}C NMR (101 MHz, DMSO-d_6) δ/ppm = 177.9 (s, CN), 164.3 (s, C, py), 159.2 (s, CN), 149.1 (s, CH, py), 139.2 (s, CH, py), 138.5 (d, ^4J(C,P)=2.5 Hz, C, ph), 132.7 (d, ^1J(C,P)=182.4 Hz, C, ph), 131.0 (d, ^2J(C,P)=14.9 Hz, CH, ph), 130.7 (d, ^3J(C,P)=9.9 Hz, CH, ph), 130.0 (s, CH, py), 123.6 (s, CH, py), 58.4 (s, $C\mathit{C}H_2N$), 44.6 (s,CH_2), 43.5 (s, CH_2), 41.0 (s, $\mathit{C}(CH_2N)_2$), 18.2 (s, CH_3), 13.6 (s, CH_3); ^{31}P NMR (162 MHz, DMSO-d_6): δ/ppm = 13.3 (s); HR-ESI-MS, m/z (MeOH), +ve: calculated for $[M]^+$ 638.0573, found 638.0565 (100%); Elemental analysis calculated for $C_{24}H_{35}Br_2CoN_5O_7P$: C 38.17%, H 4.67%, N 9.27%, P 4.10%; found: C 38.46%, H 4.55%, N 8.64%, P 4.29%; ATR-IR: 3600-2400 cm^{-1} (OH, broad), 1130 cm^{-1} (P=O); UV-vis (water): λ = 259 nm (ε = $1.864\cdot10^4$ L mol^{-1} cm^{-1}), 219 nm ($2.774\cdot10^4$ L mol^{-1} cm^{-1}).

Supporting Tables

Table S1. Comparison of ^{1}H NMR shifts of pyridine protons in diamine **4**, ^{Et}**CoP^{3}**, **CoP^{3}**, 2-picoline and to the previously reported complex [CoBr(L)]ClO_4 (L = [(DO)(DOH)(2-CH_2py)pn]).

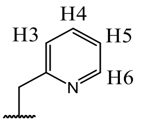

	δ / ppm<br>diamine **4**[a]	δ / ppm<br>^{Et}**CoP^{3}**[a]	δ / ppm<br>2-picoline[b,c]	δ / ppm<br>**CoP^{3}**[b]	δ / ppm<br>$[CoBr(L)]^{+}$[b,4]
**H6**	8.57	7.86-7.66	8.44	7.80-7.62	7.77
**H5**	7.20-7.13	6.98	7.17	7.26	7.27
**H4**	7.61	7.55	7.66	7.80-7.62	7.77
**H3**	7.20-7.13	7.28-7.22	7.24	7.35	7.33

[a] ^{1}H NMR spectrum recorded in $CDCl_3$; [b] ^{1}H NMR spectrum recorded in DMSO-d_6.

Table S2. I_{cat} and I_{cat}/I_p values determined for **CoP^{2}** and **CoP^{3}**.[a]

pH	I_{cat} / μA		I_{cat}/I_p	
	**CoP^{3}**	**CoP^{2}**	**CoP^{3}**	**CoP^{2}**
3	20.3	21.5	9.1	9.1
4	22.7	22.9	10	9.9
5	22.7	18.8	9	8.9[b]
6	19.9	13	7.8	6.3[b]
7	16.9	10.3	6.6	4.7[b]

[a]The I_{cat}/I_P ratio was determined for the peak current I_p of the non-catalytic Co^{III}/Co^{II} reduction at a scan rate of 20 mV s^{-1}. For I_{cat} the peak current of the reduction wave following the Co^{II}/Co^{I} reduction was determined. [b]At pH values above 4, the Co^{III}/Co^{II} couple in **CoP^{2}** becomes broad and irreversible preventing the reliable determination of I_p.

Table S3. Loading of the three **CoP^n** catalysts per geometrical surface area of ITO|*meso*ITO|**CoP^n** electrodes as determined by integrating redox waves in cyclic voltammetry (CV) traces recorded in TEOA/Na_2SO_4 (pH 7) and acetate (pH 4.5) electrolyte solution.

**Catalyst**	**TEOA/Na_2SO_4**	**acetate buffer**
	n **/ nmol cm^{-2}**	***n*** **/ nmol cm^{-2}**
**CoP^1**	21.0 ± 1.8[a]	*n.d.*[c]
**CoP^2**	34.9 ± 5.9[b]	38.4 ± 5.8[b]
**CoP^3**	23.9 ± 3.4[b]	21.2 ± 1.6[b]

[a]Mean value with standard deviation (σ) for first cathodic CV scans only; [b]Mean value with standard deviation (σ) from the first CV scans; [c]Not determined due to instability, fast desorption and broad waves in CVs.

Table S4. Photocatalytic experiments using TEOA buffer (pH 7, 0.1 M) as sacrificial electron donor. 5 mg of either TiO_2 or ZrO_2 nanoparticles were used in colloidal systems. The total volume of the solution or suspension was 2.25 mL. A 420 nm cut-off filter was used (100 mW cm^{-2}, AM 1.5G).

Entry	System	n (**CoP^n**) / µmol	n (**RuP**) / µmol	TOF (1 h) / mol H_2 (mol catalyst)$^{-1}$ h^{-1}	TON (4 h) / mol H_2 (mol catalyst)$^{-1}$	n (H_2) / µmol (4 h)
1	**RuP**|TiO_2|**CoP^3**	0.025	0.1	6.2 ± 0.7	12.8 ± 2.3	0.32 ± 0.06
2	**RuP**|TiO_2|**CoP^3**	0.05	0.1	9.2 ± 0.4	12.5 ± 1.6	0.62 ± 0.08
3	**RuP**|TiO_2|**CoP^3**	0.1	0.1	10.3 ± 0.4	12.3 ± 0.3	1.23 ± 0.03
4	**RuP**|TiO_2|**CoP^3**	0.2	0.1	9.5 ± 0.2	16.5 ± 0.5	3.29 ± 0.10
5	**RuP**|TiO_2|**CoP^3**	0.1	0.05	5.7 ± 0.6	8.6 ± 1.5	0.86 ± 0.15
6	**RuP**|TiO_2|**CoP^3**	0.1	0.2	9.7 ± 0.4	22.0 ± 1.5	2.20 ± 0.15
7	**RuP**|TiO_2|**CoP^3**	0.1	0.3	8.7 ± 0.4	19.8 ± 0.2	1.98 ± 0.02
8	**RuP**|ZrO_2|**CoP^3**	0.1	0.1	–	–	<0.03[a]
9	**RuP**|**CoP^3**	0.1	0.1	–	–	<0.03[a]
10	**RuP**|TiO_2|**CoP^2**	0.1	0.1	0.6 ± 0.02	2.4 ± 0.1	0.24 ± 0.01
11	**RuP**|TiO_2|**CoP^1**	0.1	0.1	44.0 ± 0.9	56.6 ± 2.2	5.66 ± 0.22

[a]Below the limit of detection by gas chromatography.

Table S5. Photocatalytic control experiments using TEOA buffer (pH 7, 0.1 M) as sacrificial electron donor. 5 mg of either TiO_2 or ZrO_2 nanoparticles were used in colloidal systems. The total volume of the solution or suspension was 2.25 mL. A 420 nm cut-off filter was used (100 mW cm^{-2}, AM 1.5G).

Entry	System	n (**CoP^3**) / µmol	n (**RuP**) / µmol	n (H_2) / µmol (4h)
12	**RuP**|TiO_2, no catalyst	–	0.1	0.14 ± 0.07[a]
13	**RuP**|TiO_2, no catalyst	–	0.2	0.38 ± 0.01[a]
14	**CoP^3**|TiO_2, no dye	0.1	–	<0.03[b]
15	**RuP**|TiO_2|$CoBr_2$[c]	–[c]	0.1	0.37 ± 0.15[a]
16	TiO_2, no dye, no catalyst, no filter	–	–	0.42 ± 0.15
17	water (no e- donor)	0.1	0.1	<0.03[b]
18	**RuP**|TiO_2|**CoP^3**, pre-loaded	–[d]	–[d]	0.74 ± 0.27
19	**RuP**|TiO_2|**CoP^3**, no light	0.1	0.1	<0.03[b]
20	no light (3h), then light (2h)	0.1	0.1	1.00 ± 0.02 (2h)[e]

[a]No H_2 was detected in the first hour of irradiation; [b]Below the limit of detection by gas chromatography; [c]0.1 µmol of $CoBr_2$ (1mM in water) were added; [d]Values not determined, [e]If sample is irradiated for 2 h directly after assembly 1.19 ± 0.03 µmol of H_2 were observed.

Table S6. Photocatalytic experiments without UV filter (100 mW cm^{-2}, AM 1.5G) using TEOA buffer (pH 7, 0.1 M) as sacrificial electron donor. 5 mg of either TiO_2 or ZrO_2 nanoparticles were used in colloidal systems. The total volume of the solution or suspension was 2.25 mL.

Entry	System	n (**CoP^3**) / µmol	TOF (1 h) / h^{-1}	TON (4 h) / mol H_2 (mol catalyst)$^{-1}$	n (H_2) / µmol (4 h)
21	TiO_2|**CoP^3**	0.1	4.9 ± 0.4	17.2 ± 1.3	1.72 ± 0.13
22	TiO_2|**CoP^3**, 25 mM phosphate	0.1	1.0 ± 0.1	3.4 ± 0.2	0.34 ± 0.02
23	TiO_2|**CoP^3**, 50 mM phosphate	0.1	0.1 ± 0.1	0.6 ± 0.1	0.06 ± 0.01

Table S7. Photocatalytic experiments using ascorbic acid (pH 4.5, 0.1 M) as sacrificial electron donor. 5 mg of either TiO_2 or ZrO_2 nanoparticles were used in colloidal systems. The total volume of the solution or suspension was 2.25 mL. A 420 nm cut-off filter was used (100 mW cm^{-2}, AM 1.5G).

Entry	System	n (**CoP**^{n}) / μmol	n (**RuP**) / μmol	TOF (1 h) / h^{-1}	TON (4 h) / mol H_2 (mol catalyst)$^{-1}$	lag phase / h	TON (total) / mol H_2 (mol catalyst)$^{-1}$	n (H_2) / μmol (4 h)
24	**RuP**|TiO_2|**CoP^3**	0.05	0.1	6.7 ± 0.6	8.3 ± 0.1	–[a]	–[b]	0.41 ± 0.01
25	**RuP**|TiO_2|**CoP^3**	0.1	0.1	12.8 ± 0.6[c]	18.4 ± 0.5	<0.5	19.1 ± 0.4	1.84 ± 0.05
26	**RuP**|TiO_2|**CoP^3**	0.2	0.1	5.0 ± 0.5[c]	1.0 ± 0.8	3	40.6 ± 6.0	0.19 ± 0.15
27	**RuP**|ZrO_2|**CoP^3**	0.05	0.1	4.4 ± 0.6	6.5 ± 0.3	–[a]	–[b]	0.33 ± 0.01
28	**RuP**|ZrO_2|**CoP^3**	0.1	0.1	8.1 ± 2.2[c]	9.9 ± 0.2	–[a]	–[b]	0.99 ±0.02
29	**RuP**|ZrO_2|**CoP^3**	0.2	0.1	2.9 ± 0.3[c]	5.9 ± 1.4	<2	9.4 ± 3.5	1.2 ± 0.3
30	**RuP|CoP^3**	0.05	0.1	4.9 ± 0.1	6.4 ± 0.2	–[a]	–[b]	0.32 ± 0.01
31	**RuP|CoP^3**	0.1	0.1	2.1 ± 0.6	3.1 ± 0.4	–[a]	–[b]	0.31 ± 0.04
32	**RuP|CoP^3**	0.2	0.1	10.0 ± 1.0[c]	15.8 ± 4.5	<2	–[b]	3.15 ± 0.90
33	**RuP**|TiO_2|**CoP^2**	0.1	0.1	1.2 ± 0.2	1.2 ± 0.08	–[a]	–[b]	0.12 ± 0.01
34	**RuP**|ZrO_2|**CoP^2**	0.1	0.1	1.0 ± 0.1	1.5 ± 0.06	–[a]	–[b]	0.15 ± 0.01
35	**RuP|CoP^2**	0.1	0.1	0.7 ± 0.05	1.7 ± 0.2	–[a]	–[b]	0.17 ± 0.02
36	**RuP**|TiO_2|**CoP^1**	0.1	0.1	–	–	–	–	<0.03[d]

[a]No lag period was observed; [b]Final TON_{Co} = TON_{Co} (4h); [c]TOFs are based on the maximum H_2 evolution rate after the initial lag period; [d]Below the limit of detection by gas chromatography.

Table S8. Photocatalytic control experiments using ascorbic acid (pH 4.5, 0.1 M) as sacrificial electron donor. 5 mg of either TiO_2 or ZrO_2 nanoparticles were used in colloidal systems. The total volume of the solution or suspension was 2.25 mL. A 420 nm cut-off filter was used (100 mW cm^{-2}, AM 1.5G).

Entry	System	n (**CoP**^{3}) / µmol	n (**RuP**) / µmol	n (H_2) / µmol (4 h)
37	**RuP**|TiO_2, no catalyst	–	0.1	<0.03[a]
38	**RuP**|ZrO_2, no catalyst	–	0.1	0.09 ± 0.02
39	**RuP**, no catalyst	–	0.1	<0.03[a]
40	TiO_2|**CoP**^{3}, no dye	0.1	–	<0.03[a]
41	ZrO_2|**CoP**^{3}, no dye	0.1	–	<0.03[a]
42	acetate buffer (**RuP**|TiO_2|**CoP**^{3}), no donor	0.1	0.1	<0.03[a]
43	**RuP**|$CoBr_2$[b]	–[b]	0.1	0.08 ± 0.03[c]
44	**RuP**|TiO_2|$CoBr_2$[b]	–[b]	0.1	<0.03[a]
45	**RuP**|TiO_2|**CoP**^{3}, no light	0.1	0.1	<0.03[a]
46	no light (3h), then light (3h)	0.1	0.1	1.56 ± 0.1 (3h)[d]

[a]Below the limit of detection by gas chromatography.; [b]0.1 µmol of $CoBr_2$ (1mM in water) were added; [c]No H_2 was detected in the first hour of irradiation; [d]If sample is irradiated for 3 h directly after assembly 1.81 ± 0.06 µmol of H_2 were observed.

Supporting Figures

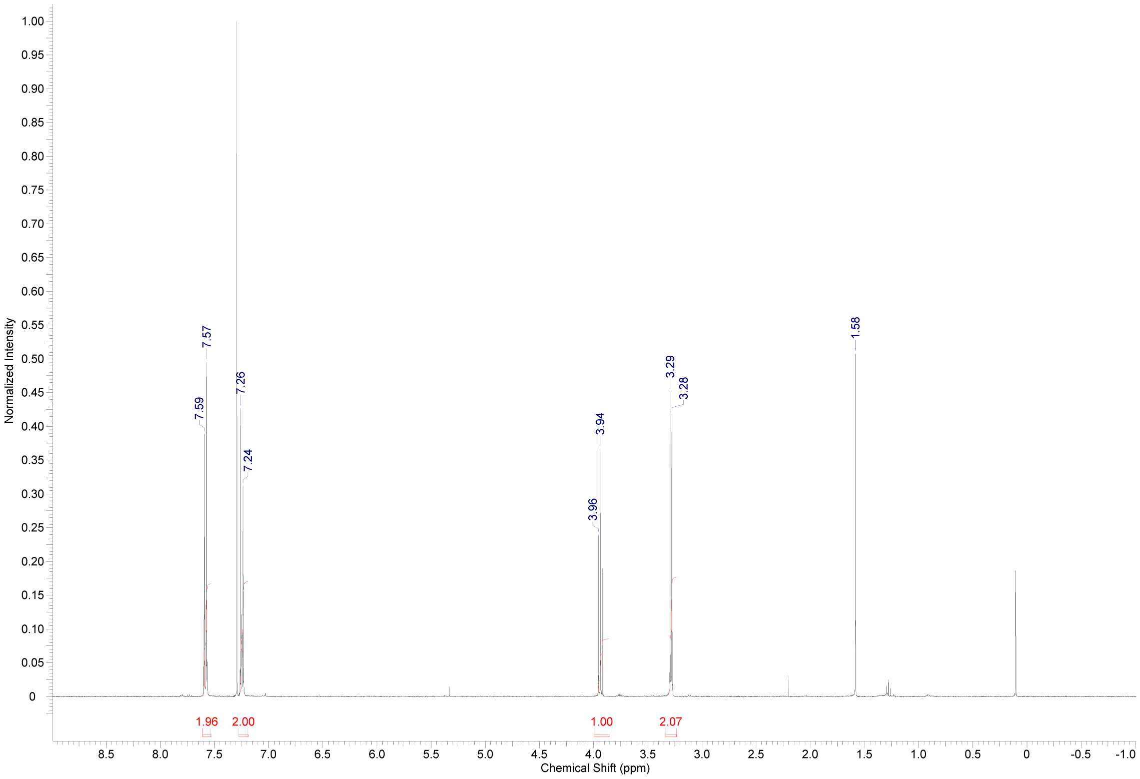

Figure S1. ^{1}H NMR spectrum of compound **1** in $CDCl_3$.

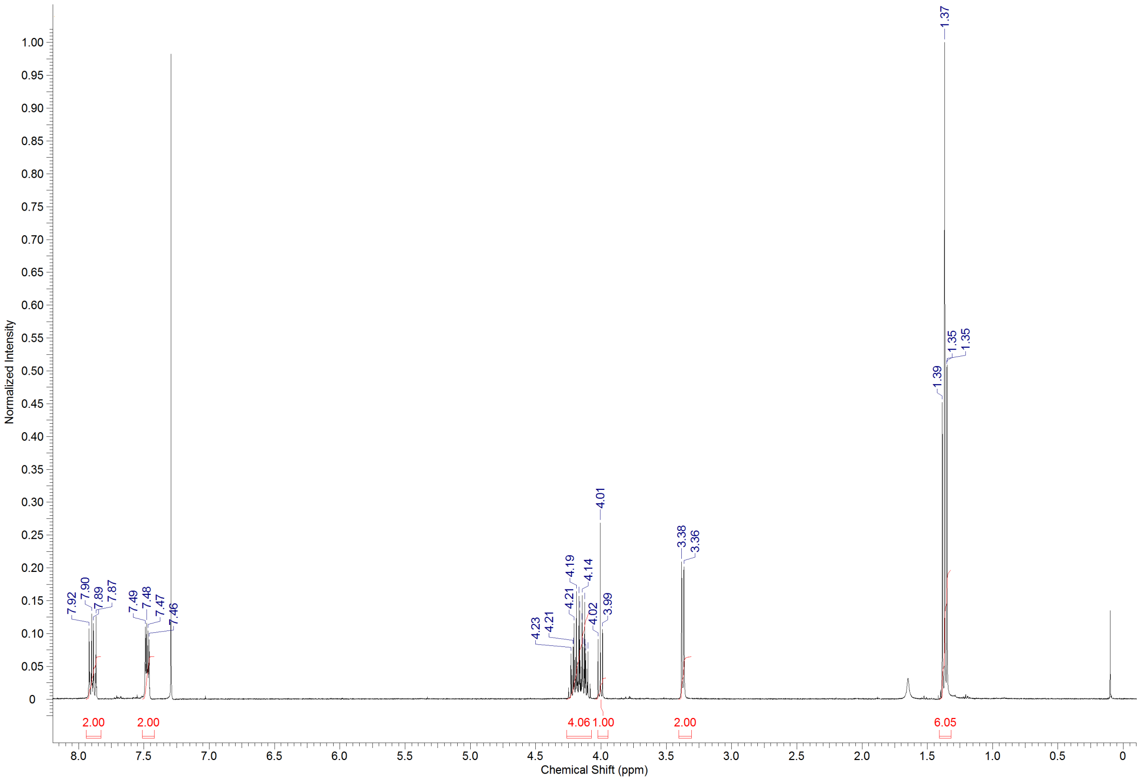

Figure S2. ^{1}H NMR spectrum of compound **2** in $CDCl_3$.

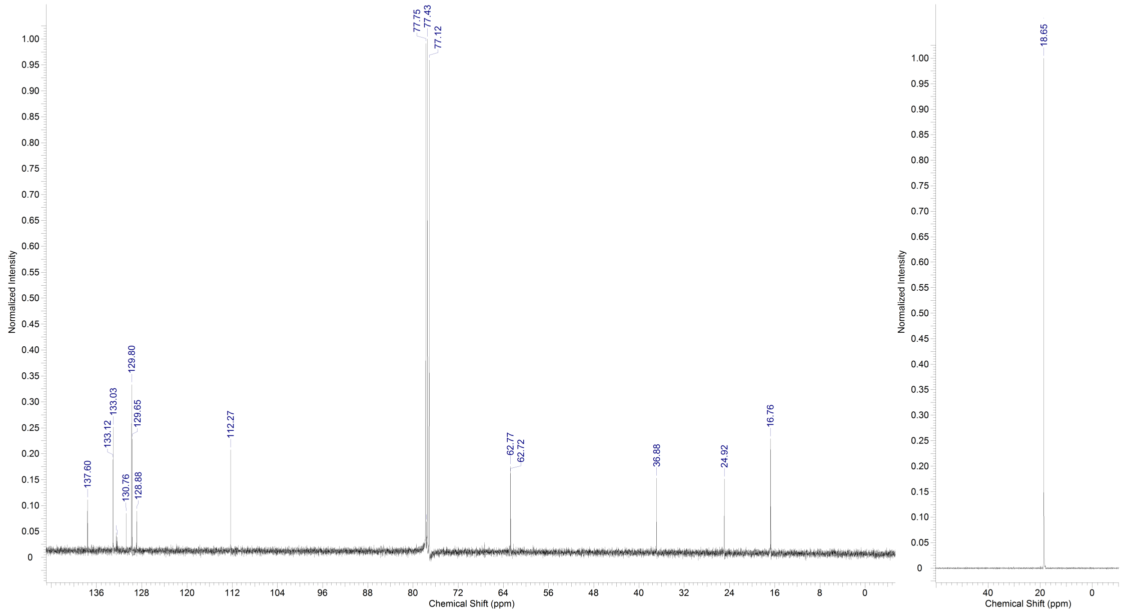

Figure S3. ^{13}C and ^{31}P NMR spectra of compound **2** in $CDCl_3$.

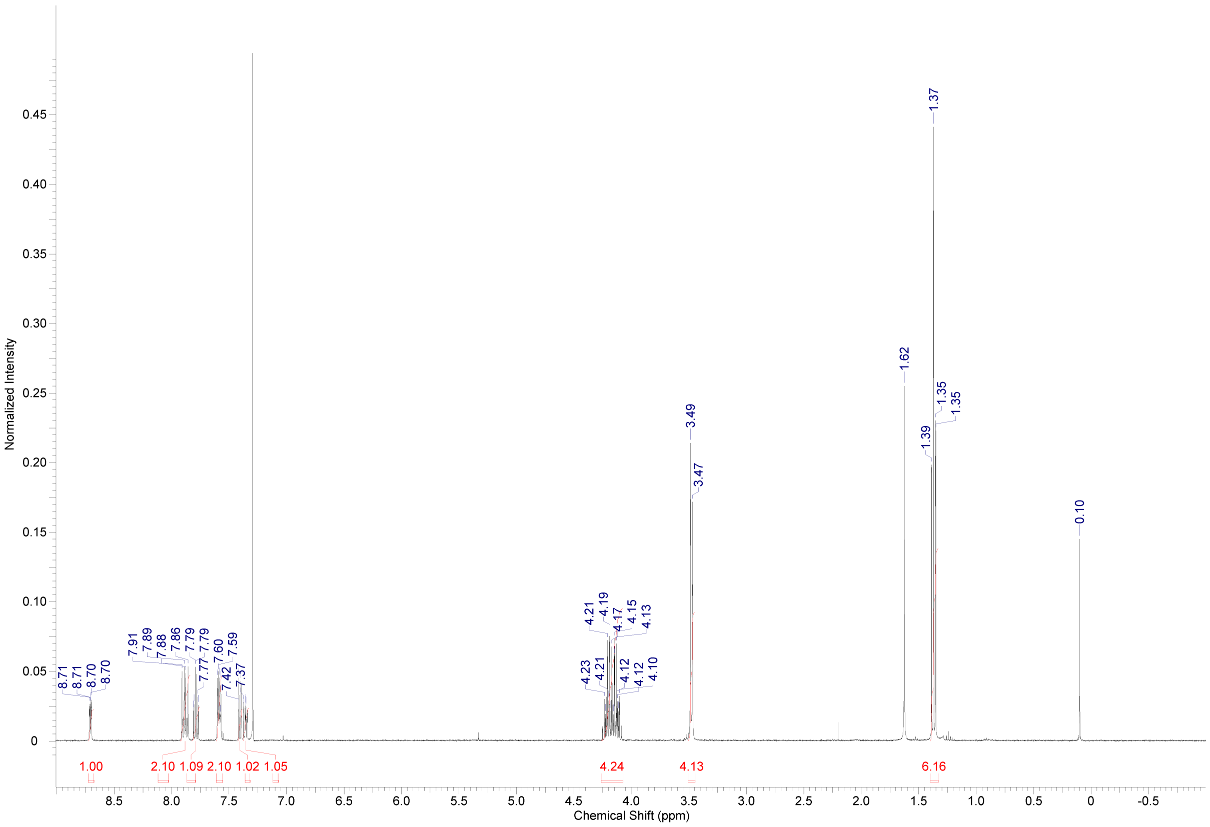

Figure S4. ^{1}H NMR spectrum of compound **3** in $CDCl_3$.

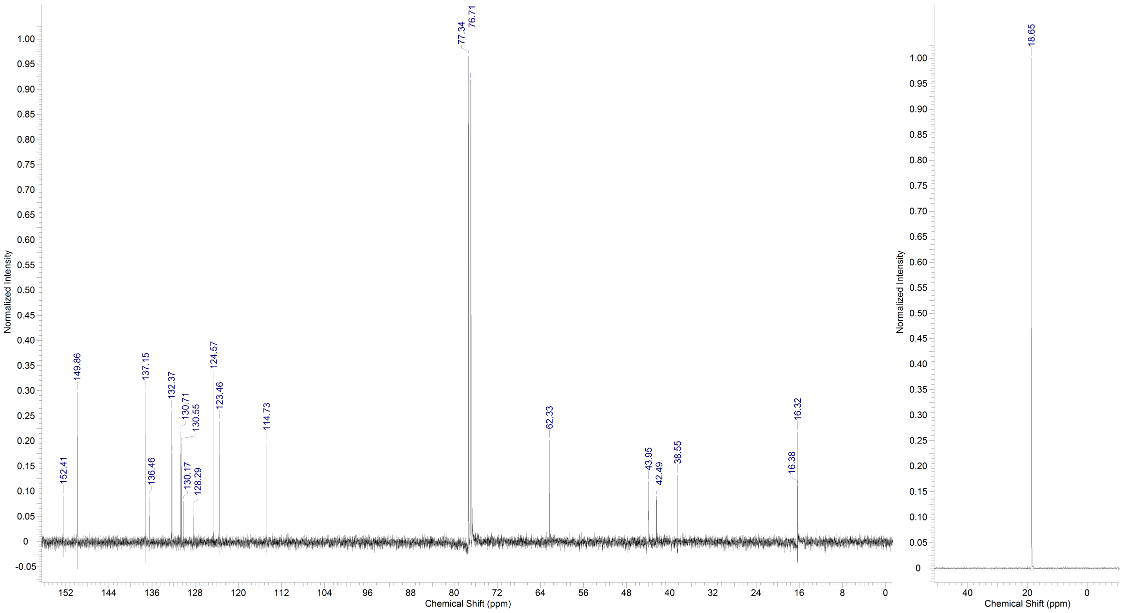

Figure S5. ^{13}C and ^{31}P NMR spectra of compound **3** in $CDCl_3$.

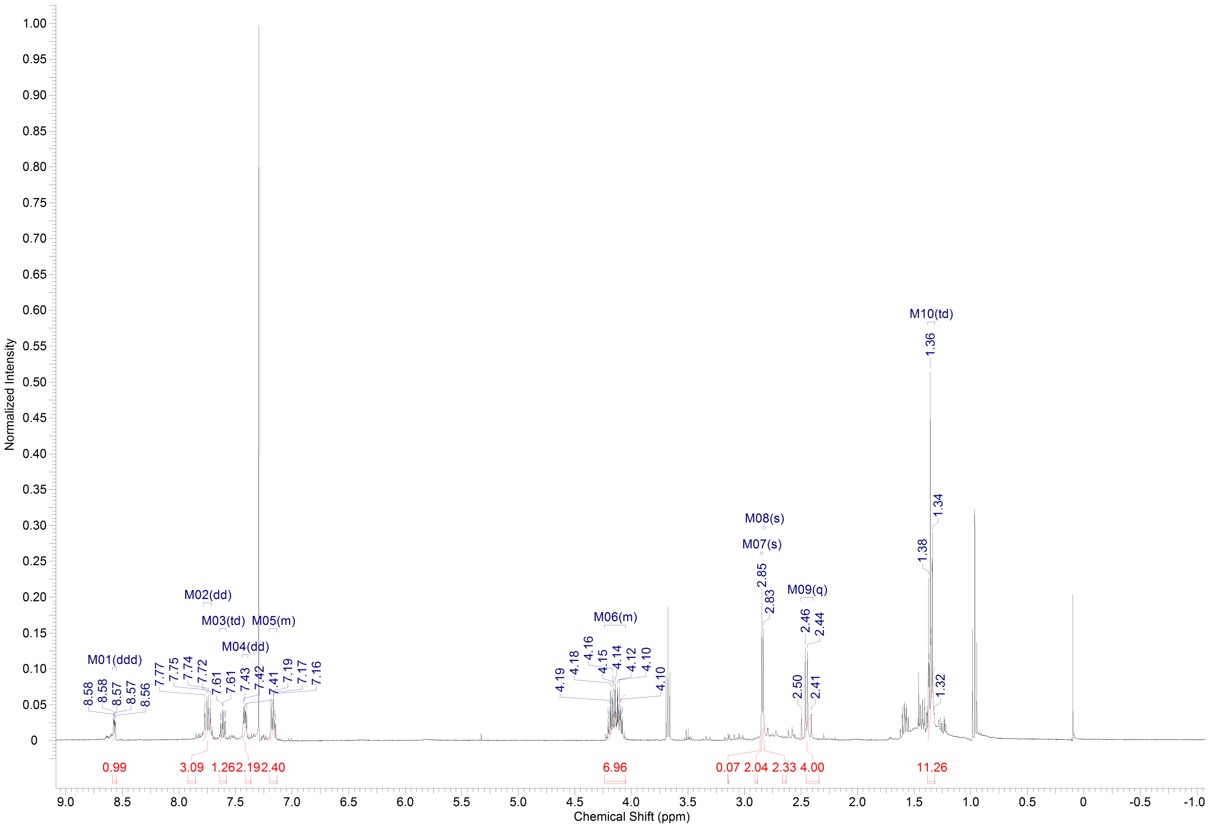

Figure S6. ^{1}H NMR spectrum of compound **4** in $CDCl_3$.

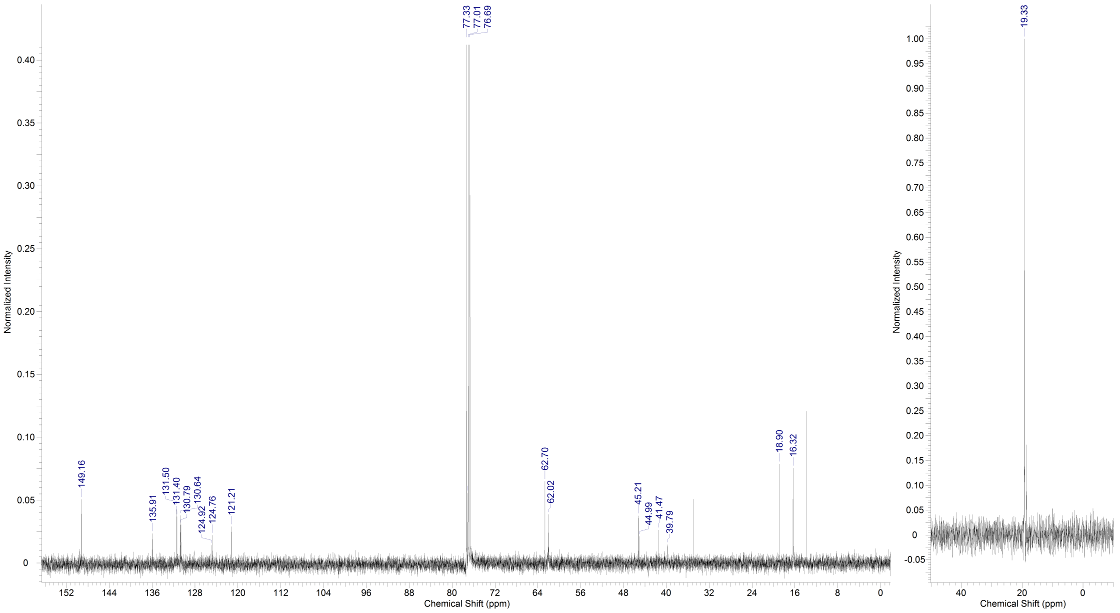

Figure S7. ^{13}C and ^{31}P NMR spectra of compound **4** in $CDCl_3$.

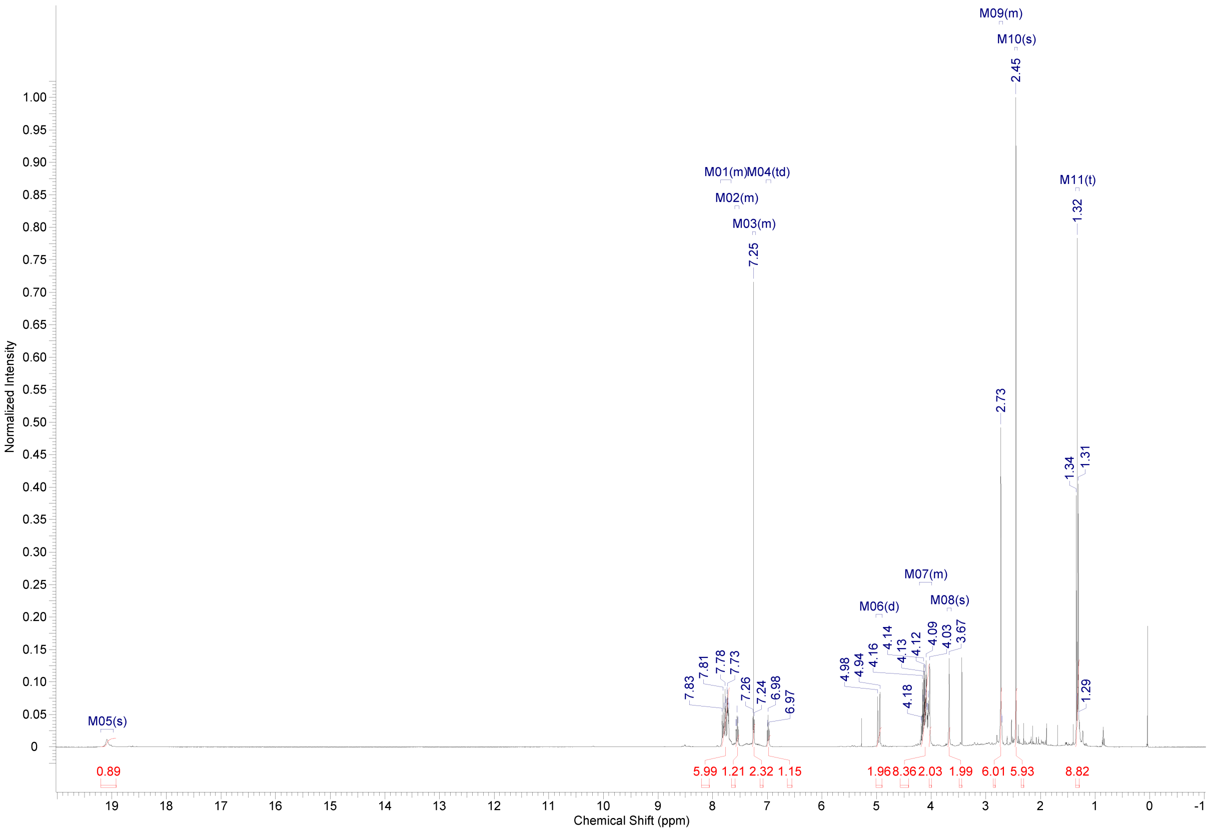

Figure S8. ^{1}H NMR spectrum of ^{Et}**CoP**^{3} in $CDCl_3$.

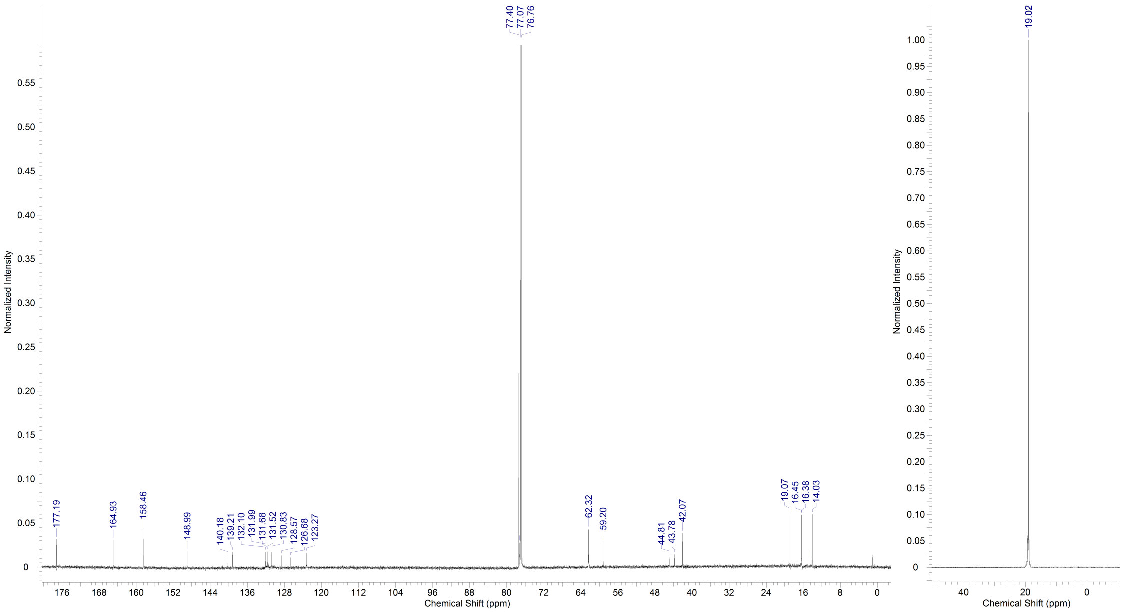

Figure S9. ^{13}C and ^{31}P NMR spectra of compound $\mathbf{^{Et}CoP^3}$ in $CDCl_3$.

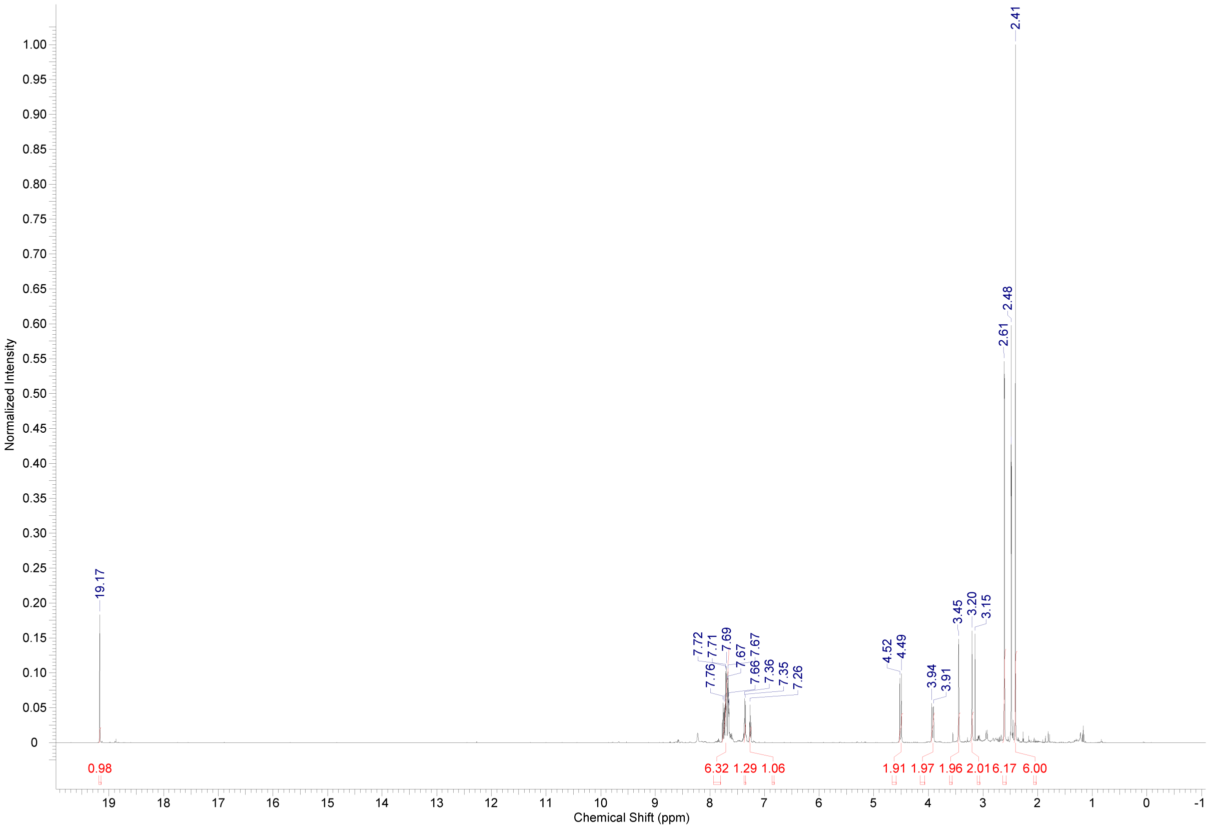

Figure S10. ^{1}H NMR spectrum of compound $\mathbf{CoP^3}$ in DMSO-d_6.

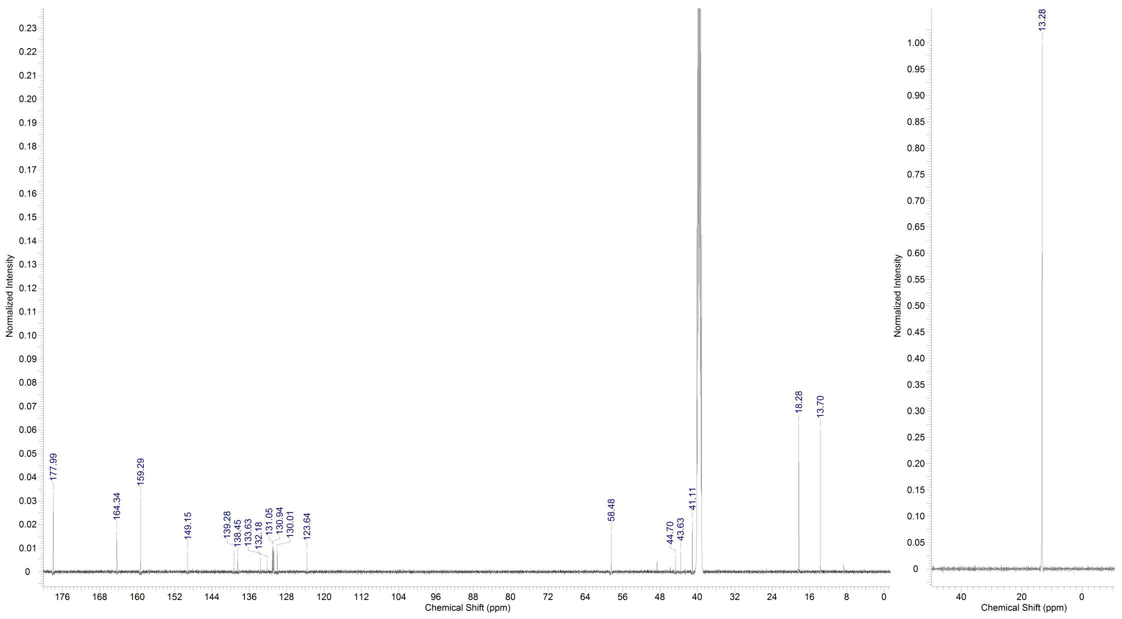

Figure S11. ^{13}C and ^{31}P NMR spectra of compound **CoP^3** in DMSO-d_6.

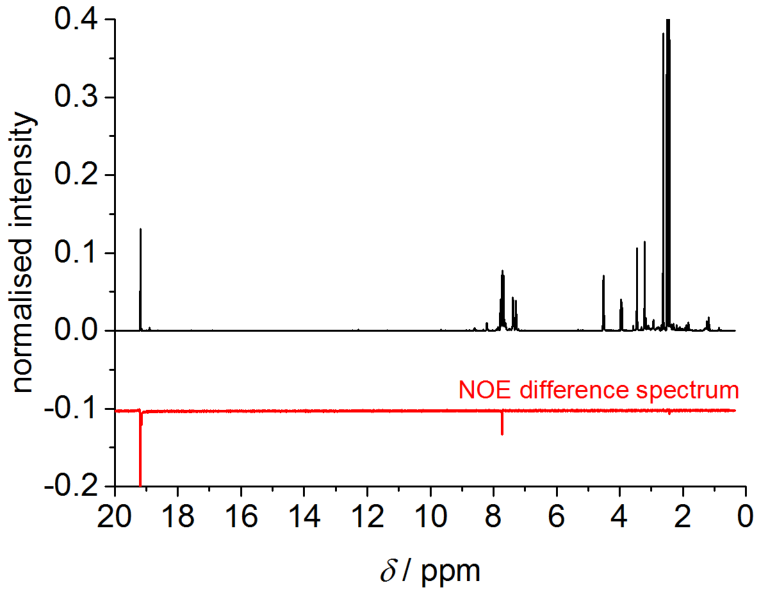

Figure S12. ^{1}H NOE spectrum of **CoP^3** in DMSO-d_6 after saturation of the signal at 19.1 ppm.

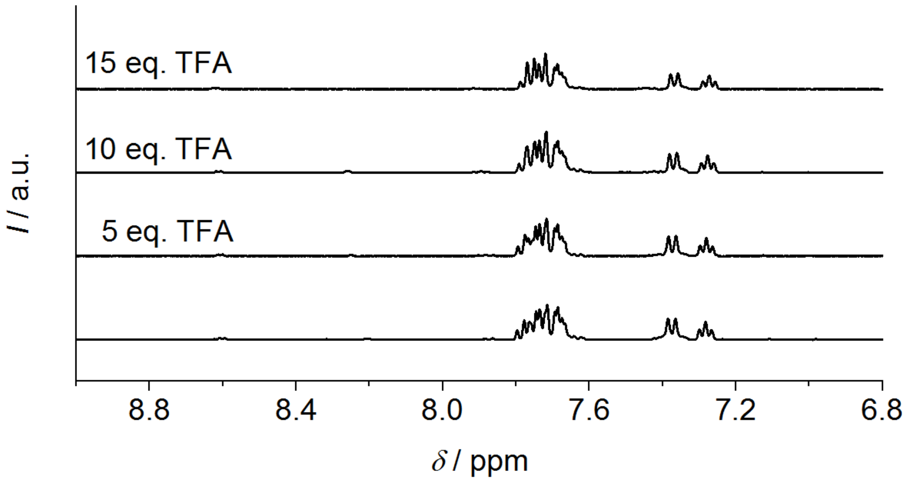

Figure S13. ^{1}H NMR spectra of **CoP^{3}** in DMSO-d_6 with addition of 0, 5, 10 and 15 eq. TFA.

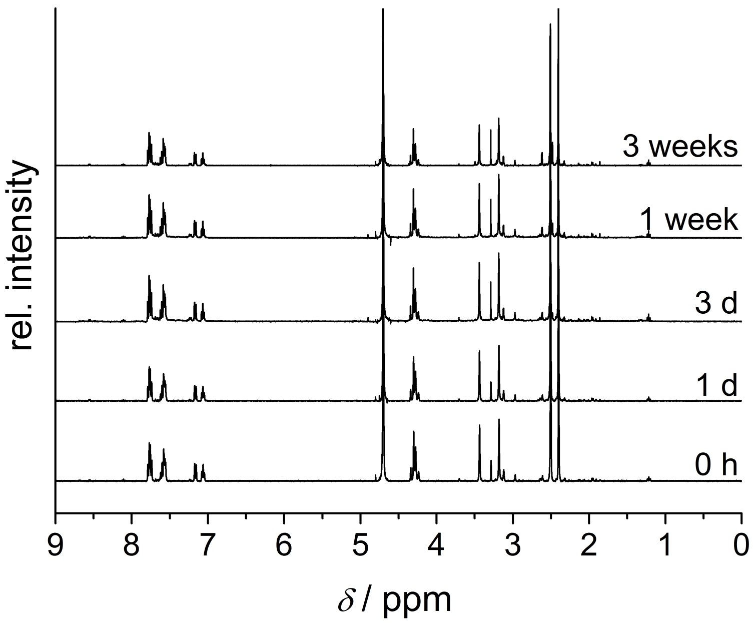

Figure S14. ^{1}H NMR spectra of **CoP^{3}** in D_2O.

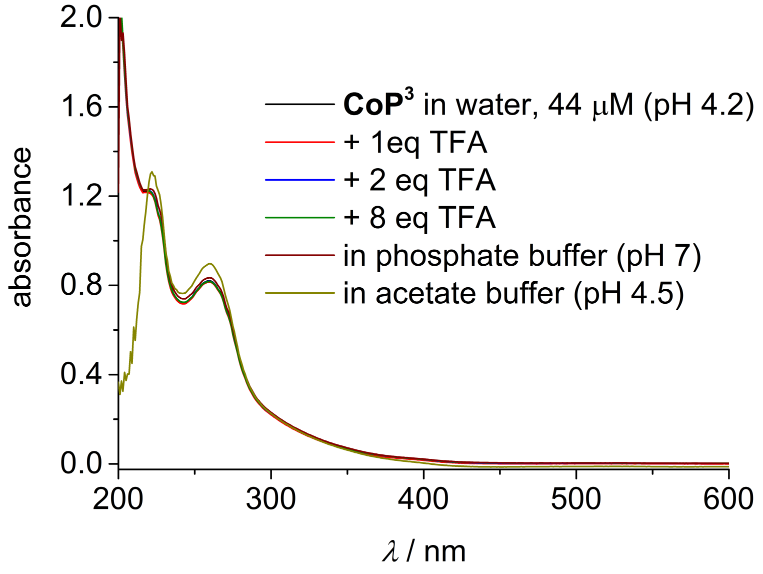

Figure S15. UV-vis spectra of **CoP**^{3} recorded in phosphate buffer (pH 7), in acetate buffer (pH 4.5) and in water (44.4 μM, pH 4.2) upon addition of 0, 1, 2 and 8 eq. of TFA.

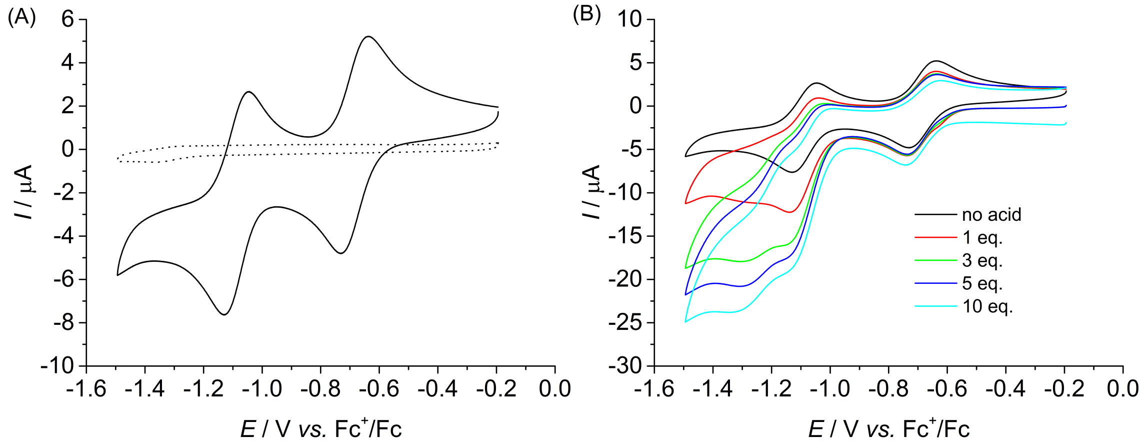

Figure S16. (A) CV scan of **CoP**^{3} (0.8 mM, solid trace) recorded in DMF/TBABF$_4$ electrolyte solution (0.1 M). The background (dashed trace) was recorded without **CoP**^{3} in solution. (B) CV scans of **CoP**^{3} (0.8 mM) upon addition of different amounts of TFA to the **CoP**^{3}-containing solution. The first scan is shown. A glassy carbon working electrode was used and CVs recorded at a scan rate of 100 mV s^{-1} and 25 °C.

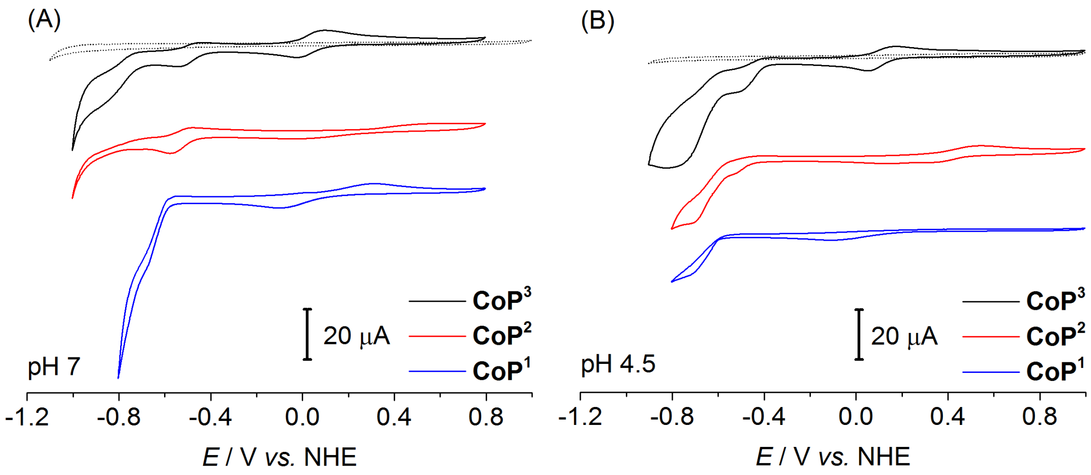

Figure S17. CVs of **CoP³** (black trace)**, CoP²** (red trace) and **CoP¹** (blue trace) in (A) pH 7 TEOA/Na_2SO_4 electrolyte solution (0.1 M each) and (B) pH 4.5 acetate buffer (0.1 M). CVs were recorded on a glassy carbon electrode with 0.8 mM catalyst in solution at a scan rate of 100 mV s^{-1}. A background scan without any catalyst in solution is shown as dotted line.

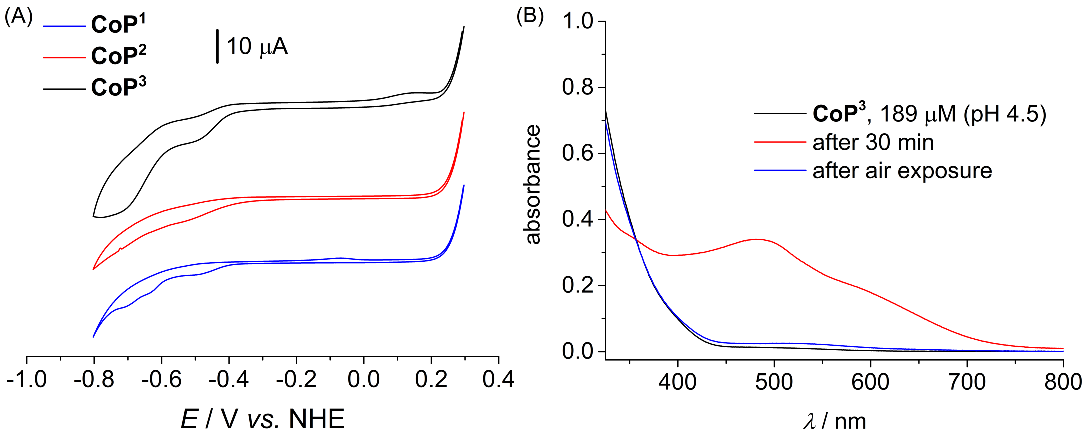

Figure S18. (A) CV scans of **CoP¹** (blue trace), **CoP²** (red trace) and **CoP³** (black trace) in ascorbic acid (0.1 M, pH 4.5, catalyst concentration: 0.8 mM), recorded at 100 mV s^{-1} and 25 °C on a glassy carbon electrode. (B) UV-vis spectra of **CoP³** (189 μM) in ascorbic acid (0.1 M, pH 4.5) after 0 min (black trace), 30 min (red trace) and after subsequent exposure to air (blue trace). The **Co^{II}P³** species features an absorption maximum at about 480 nm ($\varepsilon = 1.8\cdot10^{-3}$ L mol^{-1} cm^{-1}).

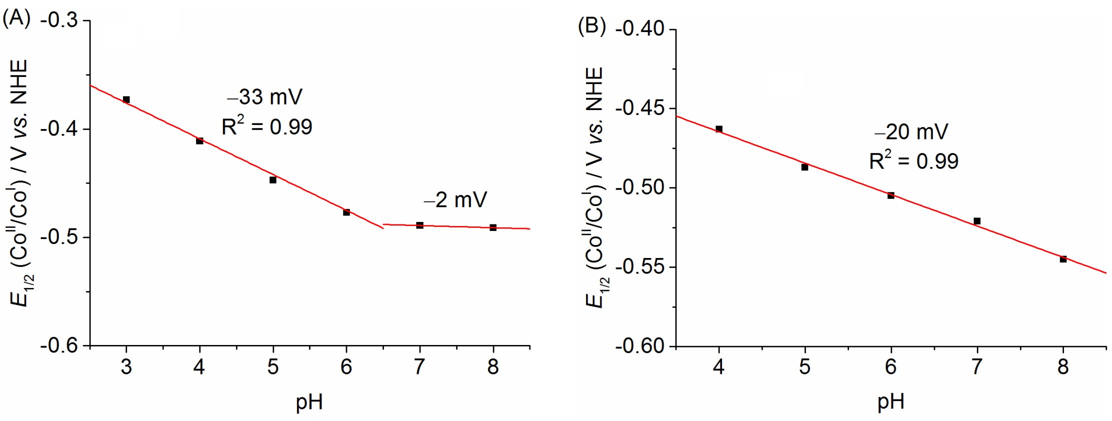

Figure S19. pH dependency of the Co^{II}/Co^{I} reduction wave ($E_{1/2}$) in (A) **CoP³** and (B) **CoP²**. The red lines represent the linear fits to the obtained data points. Values were determined from CVs recorded Britton-Robinson buffer at 20 mV s^{-1}.

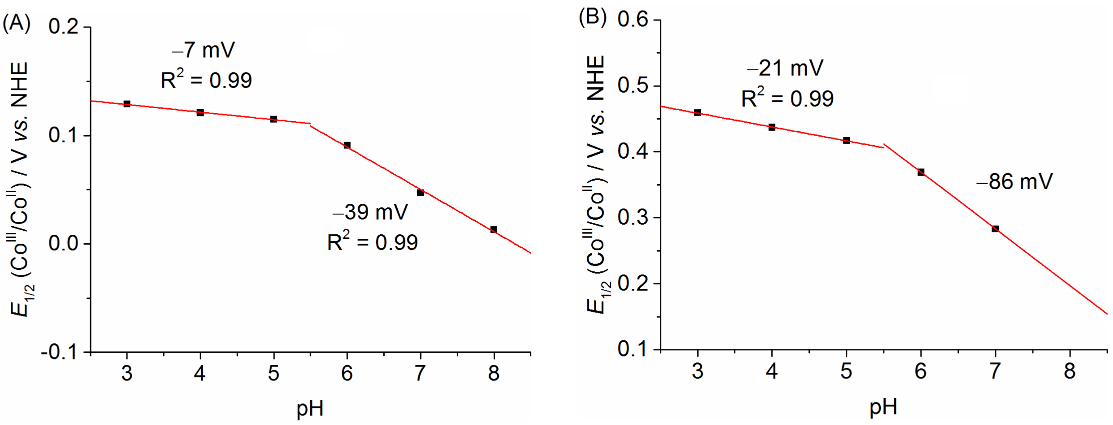

Figure S20. pH dependency of the Co^{III}/Co^{II} reduction wave ($E_{1/2}$) in (A) **CoP³** and (B) **CoP²**. The red lines represent the linear fits to the obtained data points. Values were determined from CVs recorded Britton-Robinson buffer at 20 mV s^{-1}.

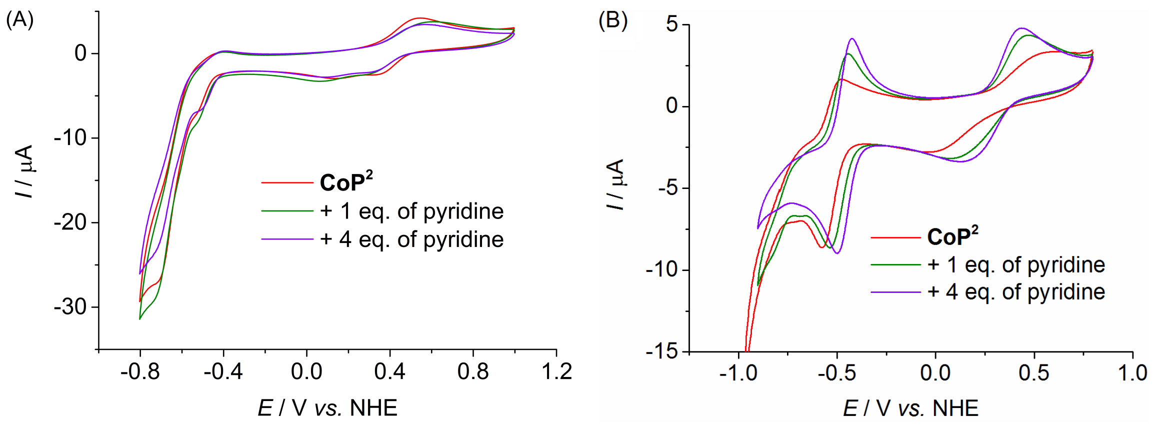

Figure S21. CV scans of $\mathbf{CoP^2}$ (0.8 mM) in (A) acetate buffer (0.1, pH 4.5) and (B) TEOA/Na_2SO_4 electrolyte (0.1 M, pH 7) upon addition of 0, 1 and 4 equivalents of pyridine. Solutions were purged for 1 min after addition of pyridine.

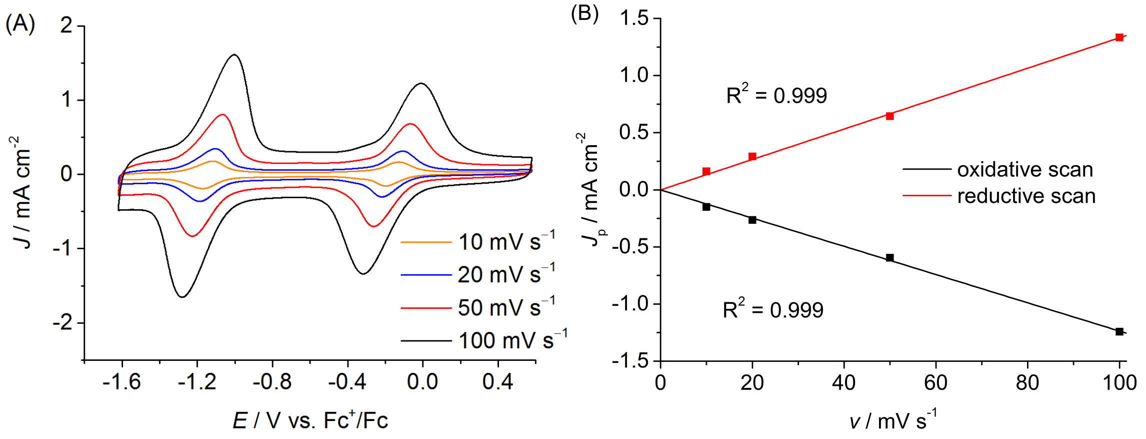

Figure S22. (A) CV scans of ITO|*meso*ITO|$\mathbf{CoP^2}$ recorded in DMF/TBABF$_4$ electrolyte at different scan rates (10, 20, 50 and 100 mV s^{-1}); (B) J_p-v dependency for ITO|*meso*ITO|$\mathbf{CoP^2}$ determined from CV scans recorded in DMF/TBABF$_4$ electrolyte (0.1 M). The black and red traces represent linear fit to the data points.

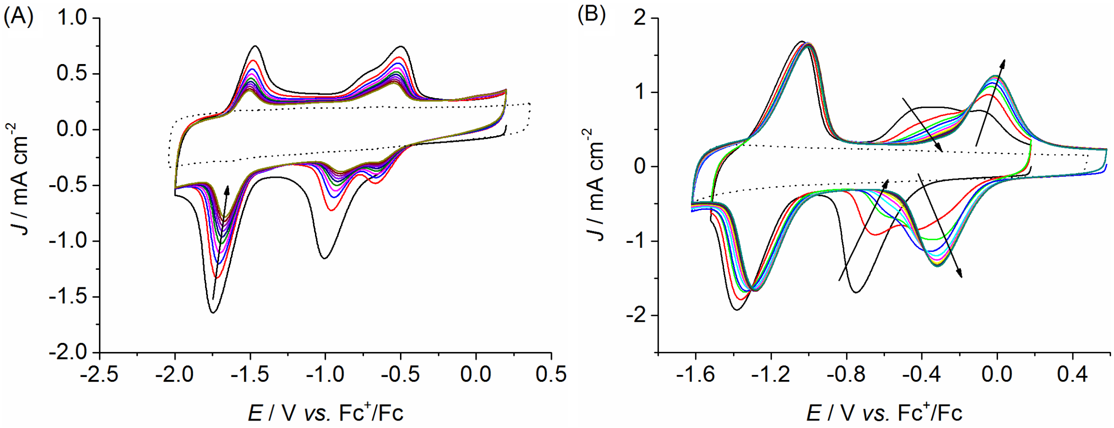

Figure S23. Consecutive CV scans of (A) ITO|*meso*ITO|**CoP[1]** and (B) ITO|*meso*ITO|**CoP[2]** in DMF/$TBABF_4$ electrolyte (0.1 M) at 100 mV s^{-1} and 25 °C. The background of ITO|*meso*ITO is shown as dotted line.

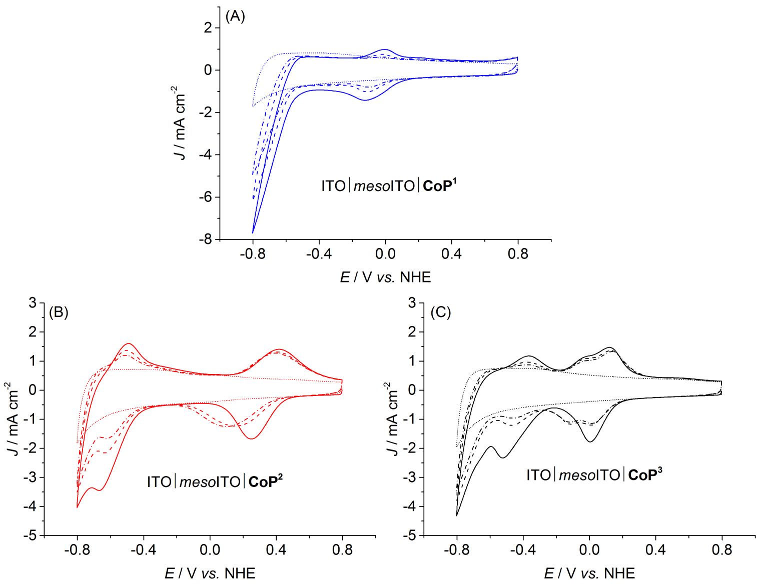

Figure S24. CV scans of (A) ITO|*meso*ITO|**CoP[1]**, (B) ITO|*meso*ITO|**CoP[2]** and (C) ITO|*meso*ITO|**CoP[3]** in pH 7 TEOA/Na_2SO_4 electrolyte (0.1 M each). CV scans were recorded at a scan rate of 100 mV s^{-1} under N_2. The first three scans are shown for ITO|*meso*ITO|**CoP[n]** electrodes. The background of ITO|*meso*ITO without cobalt catalyst is shown as dotted line.

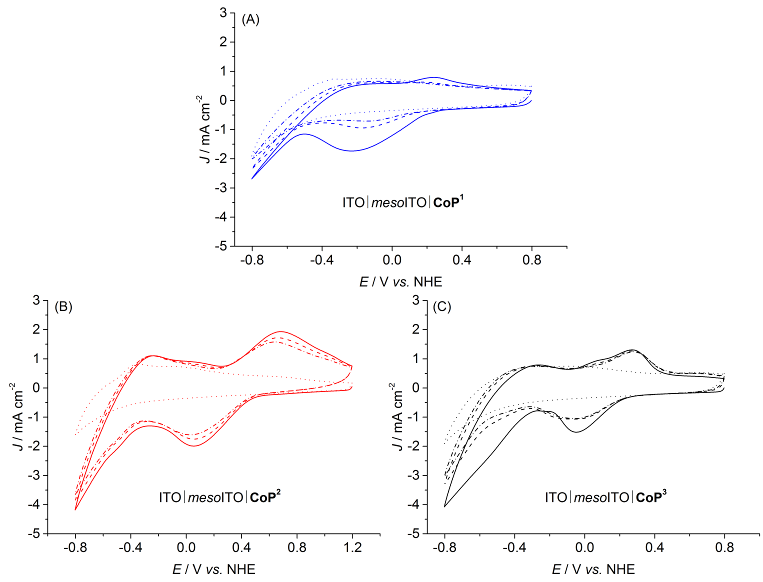

Figure S25. CV scans of (A) ITO|*meso*ITO|**CoP**^{1}, (B) ITO|*meso*ITO|**CoP**^{2} and (C) ITO|*meso*ITO|**CoP**^{3} in pH 4.5 acetate buffer (0.1 M). CV scans were recorded at a scan rate of 100 mV s^{-1} under N_2. The first three scans are shown for ITO|*meso*ITO|**CoP**^{n} electrodes. The background of ITO|*meso*ITO without cobalt catalyst is shown as dotted line.

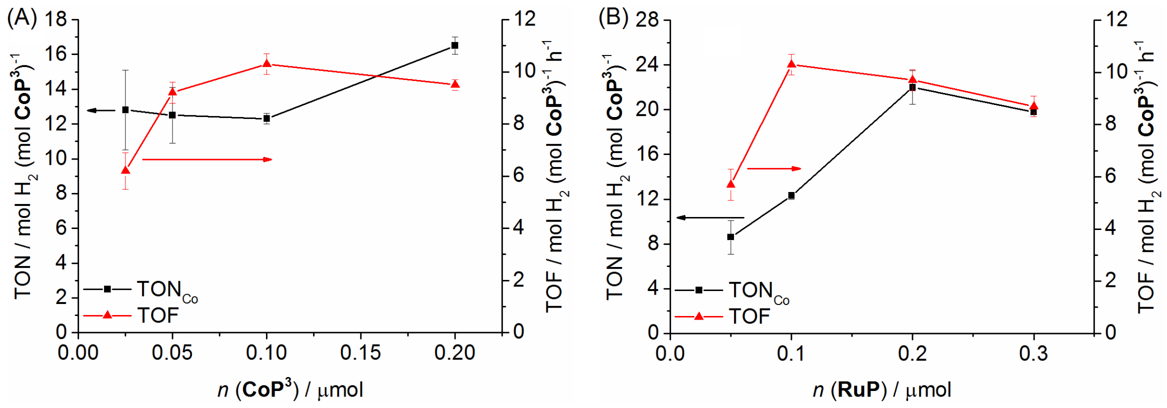

Figure S26. Changes of TON_{Co} and TOF when the (A) **CoP**^{3} or (B) **RuP** concentration is varied in **RuP**|TiO_2|**CoP**^{3} using TEOA (0.1 M, pH 7) as electron donor.

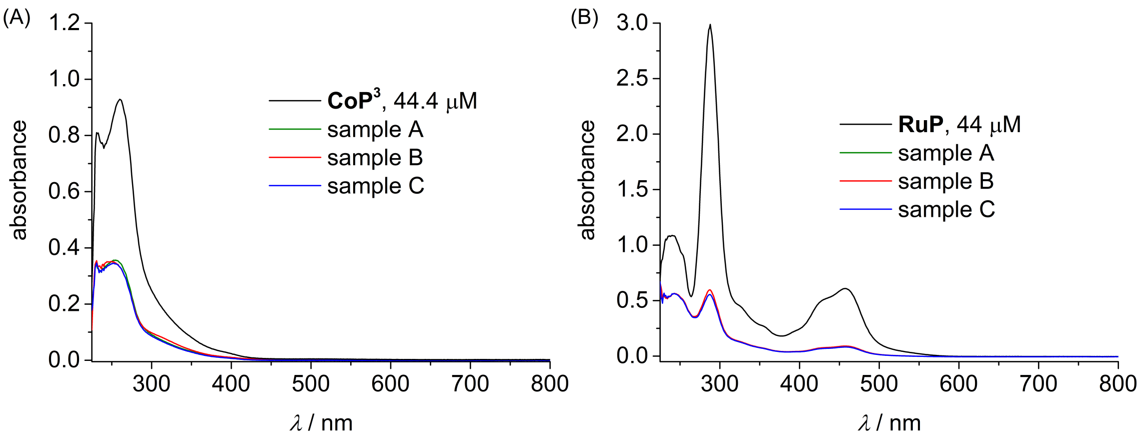

Figure S27. UV-vis spectra of supernatant after stirring 5 mg TiO_2 particles with (A) 0.1 µmol **CoP**^{3} and (B) 0.1 µmol **CoP**^{3} and 0.1 µmol **RuP** in 2.25 mL TEOA buffer (0.1 M, pH 7). About 60% of **CoP**^{3} was attached to the particles. For **RuP**, an attachment of 80 % was determined in the presence of **CoP**^{3}.

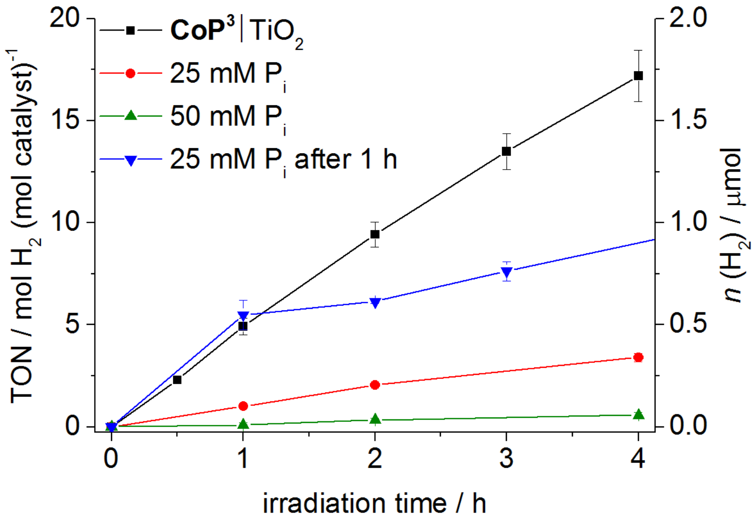

Figure S28. Photocatalytic activity (TON and H_2 produced) of TiO_2|**CoP**^{3} in pH 7 TEOA solution (0.1 M) under full spectrum irradiation (AM 1.5G, 100 mW cm^{-2}, no filter, no dye) without and with addition of phosphate buffer (25 or 50 mM, pH 7).

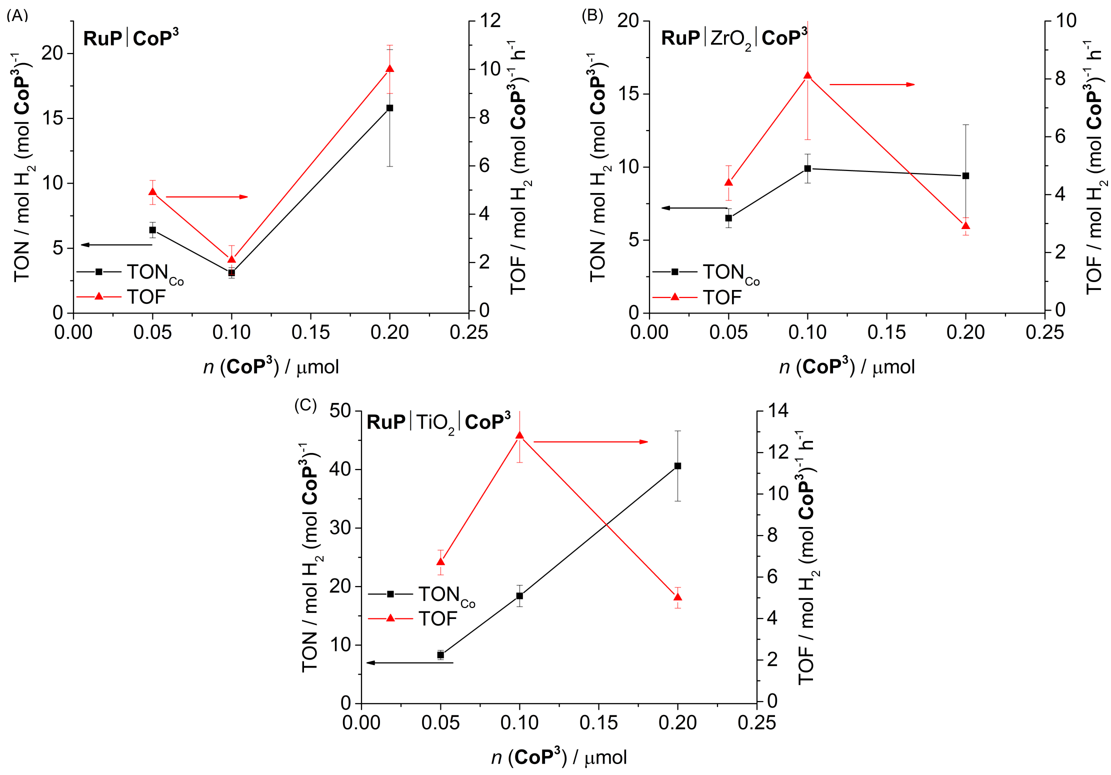

Figure S29. Observed changes of final TON_{Co} and TOF when the **CoP^3** concentration is varied in (A) **RuP|CoP^3**, (B) **RuP**|ZrO_2|**CoP^3** and (C) **RuP**|TiO_2|**CoP^3** using ascorbic acid (0.1 M, pH 4.5) as electron donor.

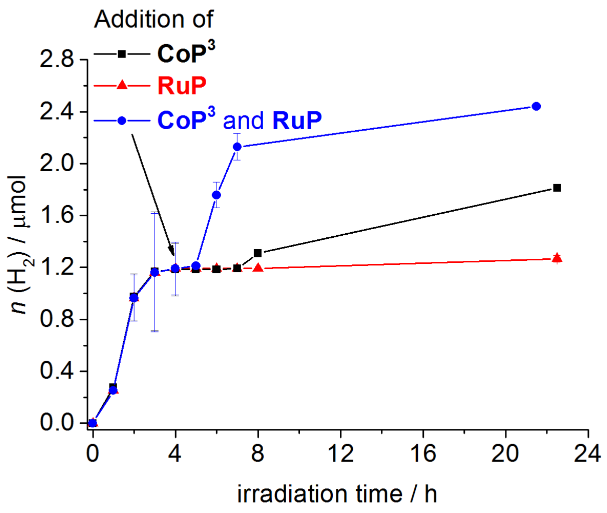

Figure S30. Reactivation of the **RuP**|TiO_2|**CoP^3** system by addition of fresh **CoP^3** (black trace), **RuP** (red trace) or both (blue trace) in ascorbic acid (0.1 M, pH 4.5).

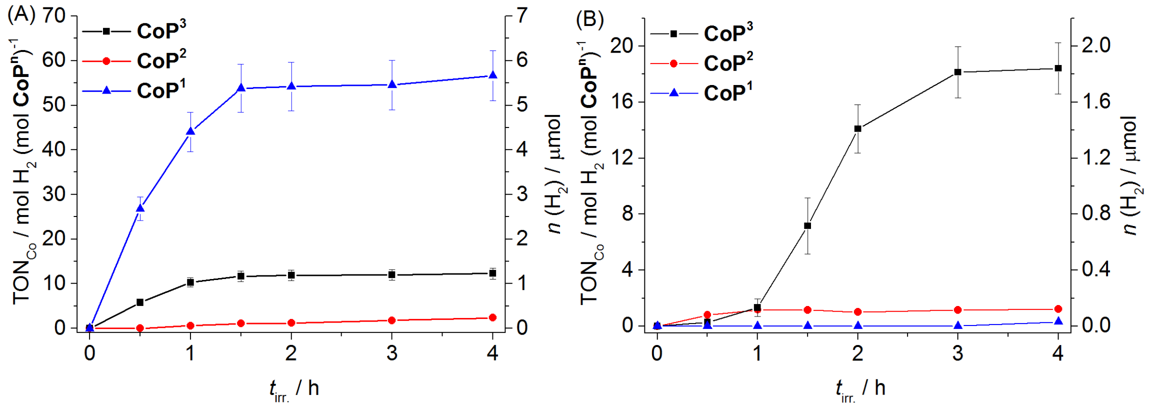

Figure S31. TON_{Co} and moles of H_2 produced in photocatalytic experiments during 4 h visible light irradiation (AM 1.5G, 100 mW cm^{-2}, $\lambda > 420$ nm) of **RuP**|TiO_2|**CoP^n** systems in (A) in 2.25 mL TEOA buffer (0.1 M, pH 7) and (B) 2.25 mL AA buffer (0.1 M, pH 4.5). A 1:1 ratio of **CoP^n** and **RuP** (0.1 µmol each) on 5 mg TiO_2 was used.

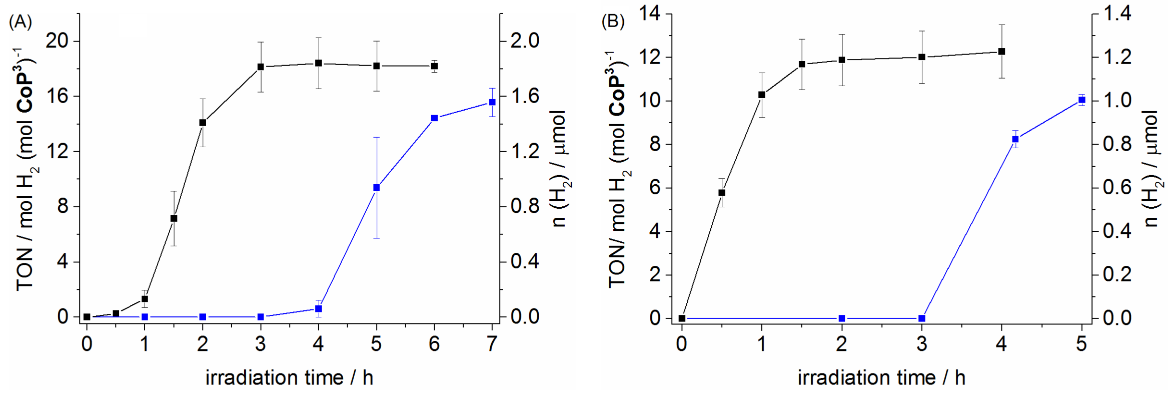

Figure S32. TON and H_2 produced in photocatalytic experiment using 0.1 µmol **CoP^3**, 0.1 µmol **RuP** and 5 mg TiO_2 in (A) 2.25 mL ascorbic acid buffer (0.1 M, pH 4.5, $\lambda > 420$ nm) and in (B) 2.25 mL TEOA buffer (0.1 M, pH 7, $\lambda > 420$ nm). Samples were kept in the dark for 3 h and then irradiated (blue trace, AM 1.5G, 100 mW cm^{-2}, $\lambda > 420$ nm). The black trace represents the photocatalytic H_2 evolution when samples were irradiated immediately after preparation. No degradation of **RuP** or **CoP^3** occurs in the dark.

Supporting References

1 (a) N. M. Muresan, J. Willkomm, D. Mersch, Y. Vaynzof and E. Reisner, *Angew. Chem. Int. Ed.*, 2012, **51**, 12749-12753; (b) F. Lakadamyali and E. Reisner, *Chem. Comm.*, 2011, **47**, 1695-1697; (c) I. Gillaizeau-Gauthier, F. Odobel, M. Alebbi, R. Argazzi, E. Costa, C. A. Bignozzi, P. Qu and G. J. Meyer, *Inorg. Chem.*, 2001, **40**, 6073-6079.

2 A. J. Bard and L. R. Faulkner, *Electrochemical Methods: Fundamentals and Applications*, John Wiley & Sons, Inc., New York, 2nd edn., 2001.

3 F. Tayyari, D. E. Wood, P. E. Fanwick and R. E. Sammelson, *Synthesis*, 2008, DOI: DOI 10.1055/s-2007-990945, 279-285.

4 L. G. Marzilli, A. Gerli and A. M. Calafat, *Inorg. Chem.*, 1992, **31**, 4617-4627.

End of Supporting Information
